# The Antimicrobial Compound Xantholysin Defines a New Group of *Pseudomonas* Cyclic Lipopeptides

**DOI:** 10.1371/journal.pone.0062946

**Published:** 2013-05-17

**Authors:** Wen Li, Hassan Rokni-Zadeh, Matthias De Vleeschouwer, Maarten G. K. Ghequire, Davy Sinnaeve, Guan-Lin Xie, Jef Rozenski, Annemieke Madder, José C. Martins, René De Mot

**Affiliations:** 1 Centre of Microbial and Plant Genetics, Department of Microbial and Molecular Systems, University of Leuven, Heverlee-Leuven, Belgium; 2 NMR and Structure Analysis Unit, Department of Organic Chemistry, Ghent University, Gent, Belgium; 3 Organic and Biomimetic Chemistry Research Group, Department of Organic Chemistry, Ghent University, Gent, Belgium; 4 State Key Laboratory of Rice Biology, Institute of Biotechnology, Zhejiang University, Hangzhou, China; 5 Laboratory of Medicinal Chemistry, Rega Institute for Medical Research, University of Leuven, Leuven, Belgium; Cornell University, United States of America

## Abstract

The rhizosphere isolate *Pseudomonas putida* BW11M1 produces a mixture of cyclic lipopeptide congeners, designated xantholysins. Properties of the major compound xantholysin A, shared with several other *Pseudomonas* lipopeptides, include antifungal activity and toxicity to Gram-positive bacteria, a supportive role in biofilm formation, and facilitation of surface colonization through swarming. Atypical is the lipopeptide’s capacity to inhibit some Gram-negative bacteria, including several xanthomonads. The lipotetradecadepsipeptides are assembled by XtlA, XtlB and XtlC, three co-linearly operating non-ribosomal peptide synthetases (NRPSs) displaying similarity in modular architecture with the entolysin-producing enzymes of the entomopathogenic *Pseudomonas entomophila* L48. A shifted serine-incorporating unit in the eight-module enzyme XtlB elongating the central peptide moiety not only generates an amino acid sequence differing at several equivalent positions from entolysin, but also directs xantholysin’s macrocyclization into an octacyclic structure, distinct from the pentacyclic closure in entolysin. Relaxed fatty acid specificity during lipoinitiation by XtlA (acylation with 3-hydroxydodec-5-enoate instead of 3-hydroxydecanoate) and for incorporation of the ultimate amino acid by XtlC (valine instead of isoleucine) account for the production of the minor structural variants xantholysin C and B, respectively. Remarkably, the genetic backbones of the xantholysin and entolysin NRPS systems also bear pronounced phylogenetic similarity to those of the *P. putida* strains PCL1445 and RW10S2, albeit generating the seemingly structurally unrelated cyclic lipopeptides putisolvin (undecapeptide containing a cyclotetrapeptide) and WLIP (nonapeptide containing a cycloheptapeptide), respectively. This similarity includes the linked genes encoding the cognate LuxR-family regulator and tripartite export system components in addition to individual modules of the NRPS enzymes, and probably reflects a common evolutionary origin. Phylogenetic scrutiny of the modules used for selective amino acid activation by these synthetases indicates that bacteria such as pseudomonads recruit and reshuffle individual biosynthetic units and blocks thereof to engineer reorganized or novel NRPS assembly lines for diversified synthesis of lipopeptides.

## Introduction

Certain genera of soil-dwelling and plant-associated bacteria, such as Streptomycetes, Myxobacteria, Bacilli, Pseudomonads, and Burkholderia, display a highly versatile secondary metabolism and, hence, have proven valuable sources for structurally very diverse metabolites with useful biological activities, including antibiotics. Whereas *Streptomyces* and other actinomycetes are major producers of clinical antibiotics, several antimicrobials from other soil bacteria, such as *Bacillus* and *Pseudomonas*, contribute to their capacity to suppress fungal plant diseases [1]. Such biocontrol effect on phytopathogenic fungi by *Pseudomonas* strains has been ascribed to *in situ* production of structurally diverse molecules: 2,4-diacetylphloroglucinol, phenazines, pyrrolnitrin, pyoluteorin, or lipopeptides [2]. Together with *Bacillus*, *Pseudomonas* are prominent producers of lipopeptides with a range of different biological activities: antagonism of microbial competitors, protection against predators, facilitation of surface motility, biofilm formation, contribution to virulence of plant pathogens, triggering of the defense response in plants [3].

Based on similarities in peptide length (ranging from 8 to 25 residues) and amino acid sequence, many of the lipopeptides produced by *Pseudomonas* strains can be assigned to specific groups, each named after a prototype compound: viscosin, amphisin, syringomycin, syringopeptin, or tolaasin [4]–[6]. The largest group with six subtypes is represented by viscosin and its analogs massetolide, viscosinamide, pseudodesmin, pseudophomin, and WLIP (white line-inducing principle). In addition, several ‘orphan’ lipopeptides not belonging to one of the known families have been described, namely putisolvin [7], orfamide [8], syringafactin [9], and entolysin [10]. Quite often a particular *Pseudomonas* strains can produce variants of a particular lipopeptide by attaching a different fatty acid or by incorporation of a similar amino acid at a certain position due to relaxed substrate specificity of the biosynthetic enzymes. With the exception of syringafactins and its analogs, cichofactins [11], all of these molecules are cyclic lipopeptides, as they contain a macrocyclic lactone ring of variable size formed between the carboxyterminal residue of the peptide and an internal amino acid (serine or threonine).

The *Pseudomonas* biosynthetic genes for syringomycin, syringopeptin, syringafactin, arthrofactin, viscosin, orfamide, massetolide, putisolvin, entolysin, WLIP, and cichofactin have been described [5], [8]–[18]. The encoded nonribosomal peptide synthetases (NRPSs) are composed of multiple modules, each consisting of an adenylation (A) domain responsible for amino acid selection and activation, a thiolation (T) domain responsible for thioesterification of the activated substrate, and a condensation (C) domain that catalyzes peptide bond formation between two amino acids. The starter condensation domain in the initiating enzyme (C1) catalyzes acylation of the first amino acid, thereby linking the lipid moiety to the oligopeptide [19], [20]. Most known *Pseudomonas* lipopeptide systems are of Type A, obeying the “co-linearity rule”. This indicates that the order and number of the NRPS modules are co-linear to the amino acid sequence of the peptide product [5], [21]. Another characteristic is the frequent occurrence of a tandem thioesterase (TE) domain in the terminating NRPS domain required for release, usually with concomitant cyclization, of the mature peptide product [6]. Multiple D-configured amino acids have been identified in *Pseudomonas* lipopeptides with known stereochemistry, but the corresponding synthetases lack standalone epimerization (E) domains, as found in for instance *Bacillus* lipopeptide NRPSs [6] or *Pseudomonas* pyoverdine synthetases [22]. This is attributed to the activity of dual C/E domains with embedded L-to-D epimerization capacity acting on an amino acid loaded on the T domain of a previous module [23].

Several *Pseudomonas* lipopeptides are harmful to fungi and oomycetes but their antibacterial activities are mostly confined to Gram-positive bacteria, while in general Gram-negative bacteria seem to be better protected by their different cell envelope architecture with a surface-exposed outer membrane [24]. It was noted, however, that a number of *Xanthomonas* species are susceptible to the cyclic lipopeptide WLIP, a member of the viscosin group, produced by *Pseudomonas putida* RW10S2 [17], *Pseudomonas fluorescens* LMG 5329 [18], and *Pseudomonas aurantiaca* PB-St2 [25]. The γ-proteobacterial genus *Xanthomonas* represents a major group of plant-associated bacteria, most of which cause plant diseases of economically important mono- and dicot crops [26], [27]. In a recent survey, three of its species were ranked among the top ten pathogenic bacteria of scientific and economic interest [28]. In plant environments, competition for nutrients and space are likely to occur between *Xanthomonas* and *Pseudomonas* as metagenomic surveys have revealed that both genera of γ-proteobacteria constitute an important fraction of the microbial communities in the rhizosphere and phyllosphere of diverse plants [29]–[32]. This report describes the characterization of *Xanthomonas*-antagonistic activity of *Pseudomonas putida* BW11M1, a strain isolated from banana rhizosphere in Sri Lanka, revealing the involvement of a new type of lipopeptide.

## Results and Discussion

### Xanthomonad-inhibitory Activity of *P. putida* BW11M1

**Figure 1:**
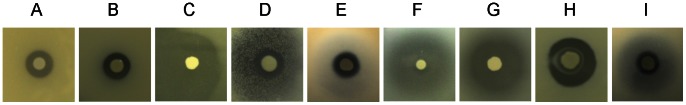
Growth inhibition of *Xanthomonas* species by *P.putida* BW11M1. Spotted BW11M1 cells (2 µl of 10^7^ CFU/ml) were overlayed with *Xanthomonas* indicator cells. (A) *X. alfalfae* subsp. *alfalfae* LMG 497; (B) *X. axonopodis* pv. *manihotis* LMG 784; (C) *X. hortorum* pv. *hederae* LMG 7411; (D) *X. sacchari* LMG 471; (E) *X. translucens* pv. *cerealis* LMG 679; (F) *X. translucens* pv. *graminis* LMG 726; (G) *X. translucens* pv. *hordei* LMG 737; (H) *X. vasicola* pv. *holcicola* LMG 736; (I) *X. vasicola* pv. *musacearum* LMG 785.

Screening of a collection of *Pseudomonas* strains isolated from tropical crop roots [33] revealed a broad xanthomonad-inhibitory activity for the banana rhizosphere isolate *P. putida* BW11M1. Using an agar diffusion assay, spotted BW11M1 cells produced a growth inhibition halo with pathovars of different *Xanthomonas* species as indicator overlay (Fig. 1). The growth inhibitory pattern for some of these strains, such as *X. translucens* pv. *cerealis* LMG 679 and *X. vasicola* pv. *musacearum* LMG 785, consists of a clear halo surrounded by a larger somewhat turbid halo, suggesting sensitivity to more than one BW11M1 metabolite. As strain BW11M1 is capable of killing pseudomonads by production of the lectin-like toxin LlpA [34], it was verified whether this bacteriocin might also be responsible for the observed growth inhibition of xanthomonads, phylogenetic relatives of pseudomonads. However, the latter antagonism was not affected in a BW11M1 *llpA* mutant [35] and an *Escherichia coli* strain producing recombinant LlpA [35] displayed no activity against *Xanthomonas* species, demonstrating LlpA not to be involved (data not shown). Furthermore, halo formation was not abolished by a non-specific protease spotted close to BW11M1 producer cells, pointing to a non-proteinaceous nature of the molecule(s) causing halo formation in the lawn of *Xanthomonas* cells (data not shown).

### Identification of *P. putida* BW11M1 Mutants Lacking Anti-Xanthomonas Activity

To identify mutants lacking anti-*Xanthomonas* activity, a *P. putida* BW11M1 plasposon mutant library [35] was screened using *X. alfalfae* subsp. *alfalfae* LMG 497 as indicator strain. Mutants lacking anti-LMG 497 activity were isolated and subjected to plasposon rescue [36] for subsequent sequencing of plasposon-flanking DNA (Table S1). The majority of the mutants (23 out of 34) was interrupted in genes showing similarity to NRPS genes, in particular to those of *Pseudomonas entomophila* L48 encoding the enzymes EtlA, EtlB and EtlC that synthesize the cyclic lipopeptide entolysin [10]. In mutant CMPG2201, a BW11M1 gene was hit that showed homology to *etlR,* the *etlA*-linked regulatory gene required for entolysin production [10]. In addition to these clustered insertions pointing to biosynthesis of a putative (lipo)peptide, additional mutations in unlinked genes were identified (Table S1). Although to be confirmed by phenotypic complementation with the respective wild-type genes, these mutations suggest that modified cell envelope characteristics (lipopolysaccharide, exopolysaccharide, large surface protein) may impair secretion of the secondary metabolite and that its production may be triggered by stress factors (*recB* and *clpB* mutants). The latter would be in line with the involvement of DnaK and ClpP in the production of the lipopeptides putisolvin by *P. putida* [37] and massetolide by *P. fluorescens* [15], respectively.

**Figure 2:**
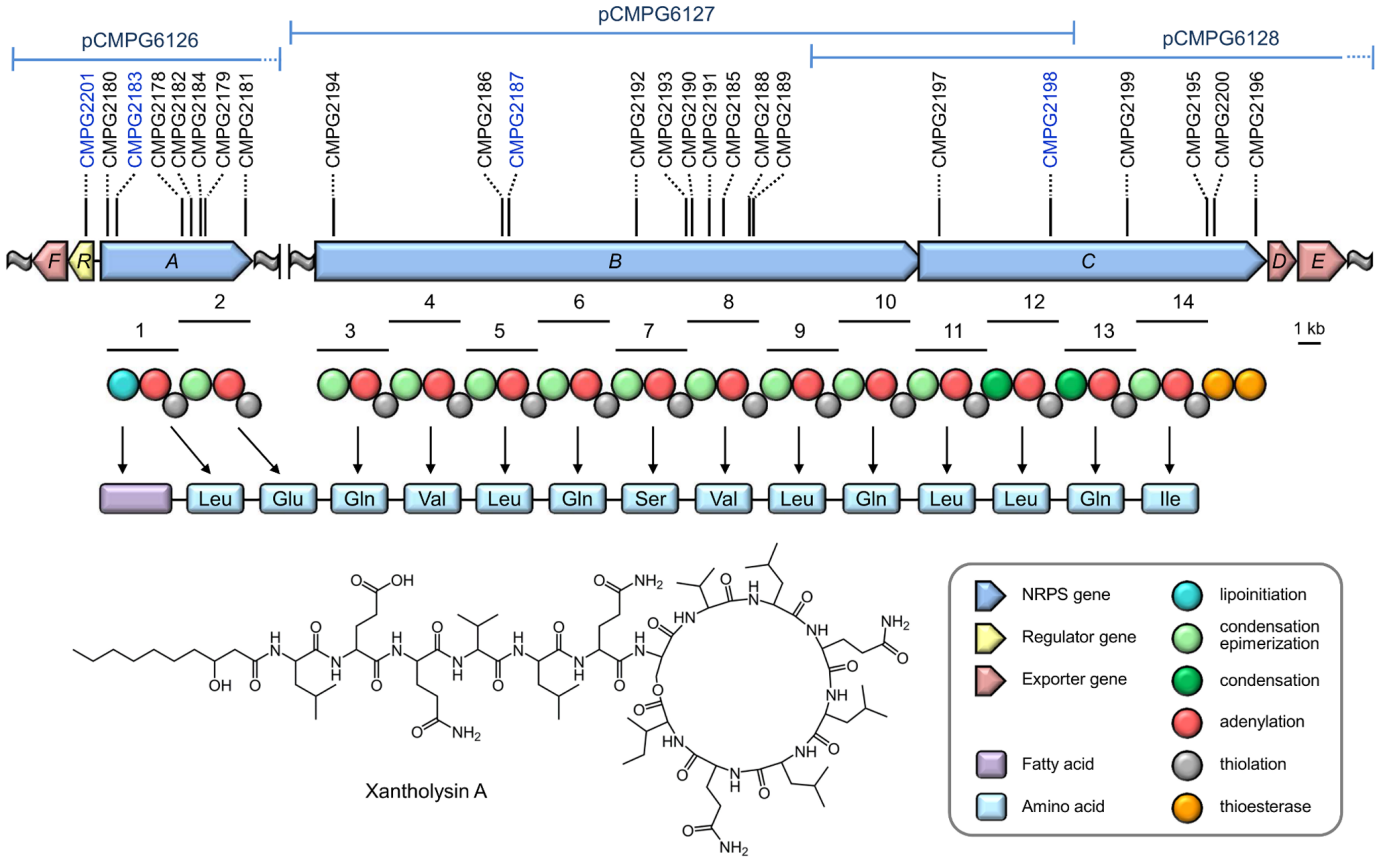
Xantholysin biosynthetic gene clusters of *P.putida* BW11M1. The organization of the genomic regions with the xantholysin synthetases genes (*xtlA*, *xtlB* and *xtlC*), the associated regulatory gene (*xtlR*) and putative export genes (*xtlD, xtlE* and *xtlF*) is shown (GenBank accession numbers: KC297505 (*xtlFRA*); KC297506 (*xtlBCDE*), together with the position of the sequenced fosmid-cloned genomic fragments. Plasposon insertion sites generating mutants without antagonistic activity *X. alfalfae* subsp. *alfalfae* LMG 497 are indicated (solid lines). Mutants selected for further phenotypic characterization are highlighted in blue font. For the encoded NRPS enzymes, the modular composition and domain architecture is visualized. The predicted amino acid sequence is based on the substrate specificity of the 14 modules, as inferred by phylogenetic analysis of *Pseudomonas* A-domain sequences (Fig. 3) and assuming consecutive co-linear biosynthesis by XtlA, XtlB and XtlC. The experimentally determined structure of the main biosynthetic product, xantholysin A, is shown.

Further analysis focused on the NRPS-related genes of *P. putida* BW11M1 apparently involved in biosynthesis of the substance inhibitory to *Xanthomonas*, tentatively designated xantholysin. In view of the anticipated large genomic DNA regions encoding multi-modular megasynthases, a fosmid library of genomic BW11M1 fragments was constructed. NRPS-specific PCR primers were designed based on the plasposon-flanking DNA sequences for the NRPS mutants listed in Table S1. Three amplicon-positive fosmids, pCMPG6126, pCMPG6127 and pCMPG6128 with estimated insert sizes of about 38 kb, 41 kb, and 40 kb respectively, were selected. Sequence analysis of a shotgun library constructed for the three pooled fosmids yielded six insert contigs that enabled the localization of three NRPS genes, designated *xtlA*, *xtlB* and *xtlC* (acronym *xtl* referring to xanthomonad-lytic activity), in two unlinked DNA regions (Fig. 2). All the plasposon insertions in the NRPS mutants are confined to these three genes. In addition, a putative regulatory gene interrupted in mutant CMPG2201, designated *xtlR*, is located upstream and oriented divergently of *xtlA*. Several *Pseudomonas* strains contain similarly organized three-membered NRPS systems consisting of an initiatory NRPS gene linked with a cognate LuxR-family gene, but located remotely from the operon with the two other NRPS genes. This genetic backbone structure was described for biosynthesis of viscosin [14], massetolide [15], entolysin [10], and WLIP [17], [18]. This pronounced analogy suggested that a non-ribosomal peptide is the likely product of the *xtl*-encoded NRPSs.

### Features of the *P. putida* BW11M1 NRPS System Required for Xanthomonas Inhibition

*In silico* analysis revealed that the three NRPSs from strain BW11M1, designated XtlA, XtlB and XtlC, comprise two, eight and four modules, respectively, each composed of a condensation (C), adenylation (A) and thiolation (T) domain (Fig. 2). A thioesterase (TE) domain tandem is located at the carboxyterminal end of XtlC. This architecture is quite similar to the *P. entomophila* biosynthetic system EtlA-EtlB-EtlC assembling the cyclic lipotetradecapeptide entolysin [10]. The level of pairwise amino acid sequence identities for XtlA/EtlA (75%), XtlB/EtlB (69%), and XtlC/EtlC (72%) further suggests that a different lipopeptide is synthesized by the sequential actions of the initiatory enzyme XtlA, XtlB and the terminating enzyme XtlC.

**Figure 3:**
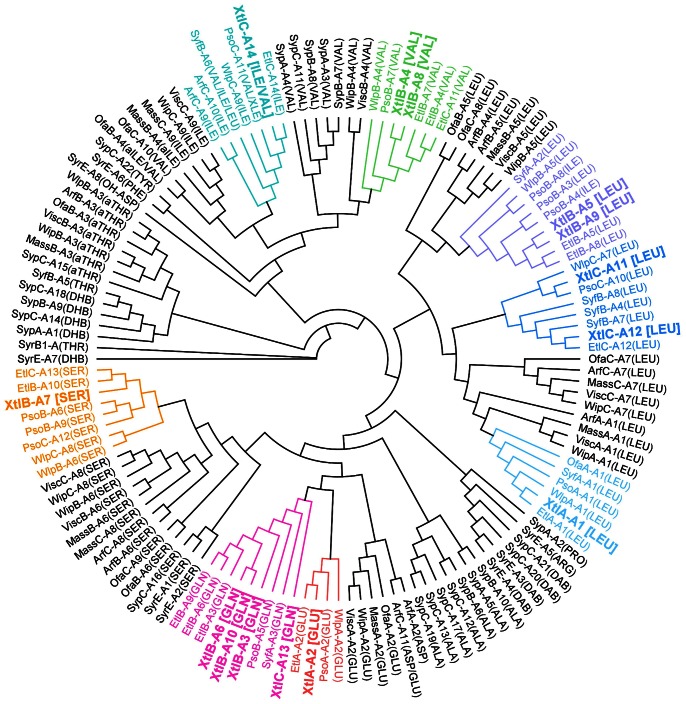
Phylogeny-based substrate specificity prediction of xantholysin synthetases. Cladogram of maximum-likelihood tree inferred from amino acid sequence alignment of adenylation (A) domains extracted from functionally characterized *Pseudomonas* NRPSs. Lipopeptide-specific codes used for NRPS enzymes: Arf (arthrofactin, *P. fluorescens* MIS38); Etl (entolysin, *P. entomophila* L48); Mass (massetolide, *P. fluorescens* SS101); Ofa (orfamide, *P. fluorescens* Pf-5); Pso (putisolvin, *P. putida* PCL1445); Syf (syringafactin, *P. syringae* DC3000); Syp (syringopeptin, *P. syringae* pv. *syringae* B301D); Syr (syringomycin, *P. syringae* pv. *syringae* strain B301D); Visc (viscosin, *P. fluorescens* SBW25); Wip (WLIP, *P. fluorescens* LMG 5329); Wlp (WLIP, *P. putida* RW10S2); Xtl (xantholysin, *P. putida* BW11M1; highlighted in larger bold font). For each domain the substrate specificity is indicated in parentheses using the standard amino acid three-letter code (for xantholysin, as determined in this work). Non-protein amino acids are annotated as follows: *allo-*threonine (aTHR); 2,3-dehydro-2-aminobutyric acid (DHB); 2,4-diaminobutyric acid (DAB); 3-hydroxyaspartate (OH-ASP); unidentified residue (Leu or Ile) in putisolvin II (XLE). Clusters comprising xantholysin domains are highlighted in different colors. The tree was rooted with the divergent SyrB1 domain.

From comparison of empirical data on the adenylating activities of NRPS enzymes it has become possible to make enzyme primary sequence-based predictions about the kind of substrate that is loaded onto a specific module. The NPRSpredictor2 tool reports the probable A-domain selectivity by comparative analysis of a 10-amino acid diagnostic motif contained within the 34-amino acid active site signature sequence ([38]; Table S2). An alternative approach consists of a phylogenetic comparison of novel A domains with those of functionally characterized NRPS systems, which was carried out here for the known *Pseudomonas* lipopeptide systems (Fig. 3). Application of both approaches suggested the following sequence for the peptide moiety in xantholysin: Leu-Glu/Asp-Gln-Val-Leu/Ile-Gln-Ser-Val-Leu/Ile-Gln-Leu-Leu-Gln-Ile/Val/Leu. The Asp residue at the second position, suggested by NRPSpredictor2 analysis (Table S2), deviates from the phylogeny-based inference (Glu). The peptide sequence of entolysin A differs from the xantholysin prediction in at least six positions (underlined): Leu-Glu-Gln-Val-Leu-Gln-Val-Leu-Gln-Ser-Val-Leu-Ser-Ile [10], [39]).

Different types of C domains can be discerned by sequence-based phylogenetic analysis [10], [15], [17], [18], [40], [41]. Application of this approach revealed that the XtlA-C1 domain clusters with known starter domains in *Pseudomonas* lipopeptide synthetases (Fig. S1), suggesting it to attach a fatty acid to the first amino acid. Only two domains (XtlC-C12, XtlC-C13) are predicted to be conventional C domains, the remaining eleven C domains being assigned to the C/E type (Fig. 2). Such quantitative predominance of C/E domains over regular C domains is a prominent feature of *Pseudomonas* lipopeptide biosynthetic systems (Fig. S1), which typically lack standalone epimerizing domains. In principal, a C/E domain epimerizes the configuration of the amino acid that is loaded onto the T domain of the previous module [23], [42]. This analysis suggests that most of the xantholysin residues would be in the D configuration. However, a certain number of deviations from such sequence-based prediction of C_α_-epimerization have been noted among *Pseudomonas* lipopeptides [8], [15], [17] and the predicted stereochemistry requires experimental validation.

The termination module of XtlC harbors two thioesterase domains (TE1/TE2 tandem) which is similar to most known *Pseudomonas* lipopeptide biosynthesis systems, except for the syringomycin group [6], [43], [44] (Fig. 2). Such thioesterase tandem also occurs in LybB from the γ-proteobacterium *Lysobacter* sp. ATCC 53042 that catalyzes release and concomitant macrocyclization of the peptide lysobactin by its TE1 domain, while TE2 serves to deacylate misprimed T domains [42]. Analysis of thioesterase domains from several *Pseudomonas* strains (Fig. S2) shows that XtlC TE1 and TE2 domains indeed form separate clusters and the pairwise similarities with the LybB domains suggest a possible thioesterase task division between product formation (TE1) and repair (TE2). The better sequence conservation among TE2 domains compared to the TE1 sequences (visualized by tighter TE2 clustering in Fig. S2) favors such general editing function. Possibly, the TE1 catalytic activity for depsi-bond formation co-evolved stronger for various peptide substrates in which lactone rings of different sizes are formed between a terminal residue and an internal serine or threonine. The hydroxylated side-chain of the sole serine residue predicted for xantholysin represents a likely candidate site for cyclization.

### Xantholysin Production is Dependent on a Cognate LuxR-family Regulator

**Figure 4:**
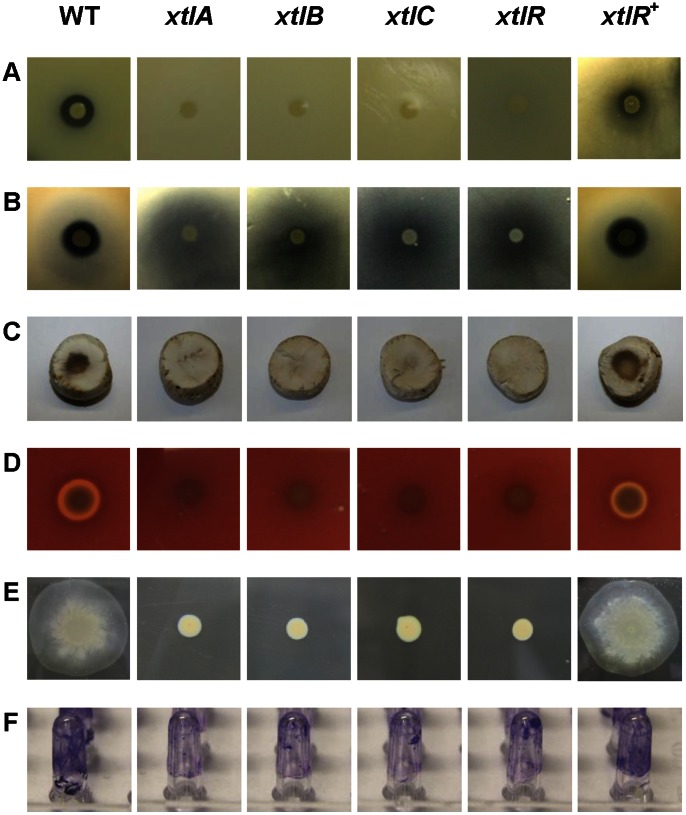
Phenotypes of *P.putida* BW11M1 and xantholysin-deficient mutants. (A) Antagonistic activity against *X. axonopodis* pv. *manihotis* LMG 784. (B) Antagonistic activity against *X. translucens* pv. *cerealis* LMG 679. (C) Formation of brown blotch on sliced *Agaricus bisporus* tissue. (D) Hemolysis on horse blood TSB agar plate. (E) Swarming on 0.8% TSB agar plate. (F) Biofilm formation on pegs visualized by staining of adherent cells. WT: BW11M1 wild type; *xtlA*, *xtlB*, *xtlC*, and *xtlR*: mutants CMPG2183, CMPG2187, CMPG2198, and CMPG2201, respectively; *xltR* ^+^: mutant CMPG2201 with pCMPG6204 containing *xtlR* of *P. putida* BW11M1. The phenotypes shown for the selected *xtlA*, *xtlB* and *xtlC* mutants are representative for the other *xtl* NRPS mutants (Table S1). The quantitative data for biofilm formation are shown in Fig. 5.

The divergently transcribed gene upstream of *xtlA*, inactivated in mutant CMPG2201 is predicted to encode a LuxR-family protein (Fig. 2). Genes encoding such LuxR-type regulators have been identified near the coding regions of most *Pseudomonas* lipopeptide-synthesizing NRPS genes [45]. These proteins, specifically controlling lipopeptide production, constitute a subfamily of regulators that lack a *N*-acylhomoserine lactone-binding domain and, consequently, are not involved in quorum sensing. The putative regulator of *P. putida* BW11M1, tentatively designated XtlR, clearly belongs to this sub-group of regulators (Fig. S3.A). With its closest homologue, EtlR of *P. entomophila* required for entolysin production [10], XtlR shares 75% amino acid identity. To confirm the involvement of XtlR in regulation of xantholysin production, mutant CMPG2201 was transformed with pCMPG6204, carrying a cloned wild-type copy of *xtlR*. The complemented mutant CMPG2201 showed anti-*Xanthomonas* activity but somewhat lower than wild-type level (Fig. 4A). A regulatory role for the equivalent genes was also reported for production of syringafactin (*syfR*; [9]), putisolvin (*psoR*; [16]), viscosin (*viscAR*; [45]), arthrofactin (*arfF*; [46]), and WLIP (*wlpR*; [17]). A downstream-positioned LuxR-type regulator gene (*viscBCR*; [45]) is also involved in viscosin production, but its counterpart (*pspto2833*) is not required for production of syringafactin [9]. No additional gene similar to *viscBCR* is present downstream of the *xtlBC* operon (data not shown), which is also the case for the *wlpBC* (WLIP) and *etlBC* (entolysin) operons [10], [17].

### Transporter Genes are Clustered with the Xantholysin Biosynthetic Genes

The genes downstream of *xtlC* (*xtlD-xtlE*) and *xtlR* (*xtlF*) potentially encode components of an export system for xantholysin (Fig. 2). This genomic organization, with equivalent genes flanking the initiating and terminating NRPS genes, is a recurrent theme for several *Pseudomonas* lipopeptide gene clusters [10], [15]–[18], [47], [48]. The XtlD-XtlE-XtlF system and its counterparts in other lipopeptide-producing pseudomonads are similar to the ATP-dependent tripartite macrolide efflux transporter MacA-MacB-TolC system of *E. coli* [49], with the OprM-like outer membrane proteins such as XtlF presumably operating as TolC equivalents in these pseudomonads [50]. These putative BW11M1 transporter components also showed the highest similarity to those of the entolysin system (Fig. S3.B). Inactivation of the Mac-like *Pseudomonas* genes downstream of the putisolvin [16], arthrofactin [47], and syringopeptin [51] biosynthetic operons cause strongly reduced but not completely abolished lipopeptide production, indicating a major but non-exclusive role in export. Such redundancy in lipopeptide export capacity may explain why no xantholysin null mutants with insertions in these putative transporter genes were identified. The involvement of an OprM homologue in lipopeptide production by *Pseudomonas* has not yet been experimentally verified.

### The Antibacterial Activity of Xantholysin is not Restricted to Xanthomonads

*X. alfalfae* subsp. *alfalfae* LMG 497 was used as an indicator strain to identify BW11M1 mutants lacking xantholysin production. To assess whether the observed inhibition of other *Xanthomonas* strains also relies on this lipopeptide system, representative anti-LMG 497 null mutants with a disrupted *xtlA*, *xtlB*, *xtlC* or *xtlR* gene, were assayed for antagonism against other *Xanthomonas* strains (Table S3). The lack of xantholysin production coincided with disappearance of the single clear halo surrounding the BW11M1 spot for sensitive strains such as *X. axonopodis* pv. *manihotis* LMG 784 (Fig. 4A). For strains such as *X. translucens* pv. *cerealis* LMG 679 with two concentric growth-inhibitory halo’s, only the smaller clear halo disappeared. The latter was restored when assaying the complemented *xtlR* mutant, also linking this phenotype to xantholysin production (Fig. 4B). With these indicator strains, the larger turbid halo remained visible but the zone of inhibition caused by the BW11M1 mutants was somewhat reduced compared to the wild type (Table S3). The sensitivity of xanthomonads to xantholysin from *P. putida* BW11M1 shows qualitative and quantitative differences compared to WLIP-mediated inhibition by *P. putida* RW10S2 [17]. For instance, growth of the strain most sensitive to WLIP, *X. citri* pv. *malvacearum* LMG 761, is only weakly affected by xantholysin production (Table S3).

To further examine the antibacterial capacity of strain BW11M1 and the role played by xantholysin production in this, an expanded indicator panel composed of Gram-negative and Gram-positive bacteria (Table S4) was tested against BW11M1 wild type and selected mutants. *P. putida* BW11M1 did not display a broad inhibitory activity among other Gram-negative bacteria, with six out of seventeen additional Gram-negative bacteria tested being inhibited by strain BW11M1: the α-proteobacteria *Azospirillum brasilense* Sp7 and *Sphingomonas wittichii* RW1, the β-proteobacteria *Burkholderia vietnamiensis* LMG 10927 and *Variovorax paradoxus* LMG 1797, and the γ-proteobacteria *Aeromonas hydrophila* ATCC 7966 and *Erwinia amylovora* CFBP 1430 (Table S3). For the *Aeromonas*, *Burkholderia* and *Erwinia* strains, xantholysin production appears to be solely responsible for inhibition. The antagonistic effect of strain BW11M1 exerted on pseudomonads, including members of the *P. syringae* group, was not abolished in the xantholysin mutants (data not shown), confirming that this is bacteriocin-mediated [34].

Growth of most of the Gram-positive bacteria strains tested (seven out of nine) was inhibited by strain BW11M1: the firmicutes *Bacillus megaterium* ATCC 13632, *Bacillus subtilis* LMG 7135 and *Staphylococcus aureus* ATCC 6358, and the actinomycetes *Mycobacterium smegmatis* DSM 43756, *Rhodococcus erythropolis* N11, *Streptomyces coelicolor* A3(2) and *Streptomyces lividans* TK24. The residual antagonistic activity towards these Gram-positive bacteria displayed by the xantholysin-deficient mutants again points to production of (an)other growth-inhibitory factor(s) by *P. putida* BW11M1 (Table S3). The antagonistic activity conferred by xantholysin production based on mutant analysis was confirmed for selected strains with purified xantholysin (purification described below; Fig. S4). When testing this sample against rice pathogen *X. oryzae* pv. *oryzae*, strains PXO99 and PXO112 proved sensitive, whereas growth of strain PXO340 was not affected (data not shown).

The inhibition of Gram-negative bacteria is a property not typically associated with lipopeptides, whereas Gram-positive bacteria are generally more susceptible [3]. In a comparative study of the antagonistic activity of five *Pseudomonas* lipopeptides (massetolide, syringomycin, orfamide, arthrofactin, and entolysin), no significant inhibition of Gram-negative bacteria was observed [24]. Conversely, among Gram-positive bacteria, an *Arthrobacter* strain (actinomycete) was susceptible to all tested compounds, except orfamide. Arthrofactin was also active against other actinomycetes (*Corynebacterium*, *Mycobacterium*).

### The Broad Antifungal Activity of *P. putida* BW11M1 is mainly Dependent on Xantholysin Production

To further assess the antimicrobial potential of xantholysin, the capacity of *P. putida* BW11M1 and its xantholysin-deficient mutants to inhibit growth of several fungi was compared. Radial mycelial outgrowth was delayed or arrested for most of the fungi confronted with a colony of wild-type BW11M1 and this was connected to xantholysin production (Fig. S5). In addition to several ascomycetes, the basidiomycete *Rhizoctonia solani* was also growth-inhibited. *P. putida* BW11M1 cells applied on the sliced fruiting body of the basidiomycete *Agaricus bisporus* [52] resulted in discoloration. This tissue damage was not visible for the xantholysin-deficient mutants, indicating it to be dependent on xantholysin production (Fig. 4C). Tissue discoloration was also observed for the complemented *xtlR* mutant CMPG2201 carrying pCMPG6204. No such blotch-like symptoms were observed following application of a BW11M1 cell suspension onto intact mushroom caps (data not shown).

Antifungal activity has been reported for several cyclic lipopeptides belonging to different structural groups [3]. These include tolaasin, a mycotoxin from the mushroom pathogen *P. tolaasii*, and its analog sclerosin [53]–[55], amphisin [56], viscosinamide [57], thanamycin (syringomycin group; [58]), and syringopeptin [59]. Immobilization and lysis of oomycete zoospores is caused by several viscosin-group members [3]. The *in vitro* antifungal activity of entolysin has not been investigated, but production of entolysin is not involved in the biocontrol activity of *P. entomophila* L48 in a *Pythium ultimum*/cucumber setup [10].

### Hemolytic Activity of *P. putida* BW11M1 is Mediated by Xantholysin

Hemolytic activity has been reported for a number of lipopeptides [10], [17], [48], [54]. This phenotype can be readily visualized by formation of a discoloured halo surrounding a colony grown on blood agar. *P. putida* BW11M1 also shows hemolytic activity, whereas mutants deficient in anti-*Xanthomonas* activity are devoid of it (Fig. 4D). In the complemented *xtlR* mutant CMPG2201 carrying pCMPG6204, hemolysis was restored, in parallel with anti-*Xanthomonas* activity (Fig. 4A, 4B), thus identifying xantholysin as the hemolytic factor produced by strain BW11M1. Hemolysis by the structurally related lipopeptide entolysin does not contribute to virulence of its entomopathogenic producer [10].

### Xantholysin is Required for Swarming and Contributes to Biofilm Formation

For several *Pseudomonas* strains the involvement of lipopeptides in swarming and biofilm formation has been reported [3], [17], [18], [48]. It was investigated whether these phenotypes are equally associated with xantholysin production. The *P. putida* BW11M1 mutants impaired in anti-*Xanthomonas* antagonism were also deficient in swarming ability on soft agar (0.8%), compared to the strong surface translocation capacity of the wild-type strain (Fig. 4E). Swarming of the regulatory mutant (CMPG2201), carrying a cloned *xtlR* copy (pCMPG6204), was comparable to the wild-type behavior (Fig. 4E). This reveals a primary role for xantholysin in swarming by *P. putida* BW11M1. Swarming capacity is also affected in *P. entomophila* mutants lacking entolysin production [10].

**Figure 5:**
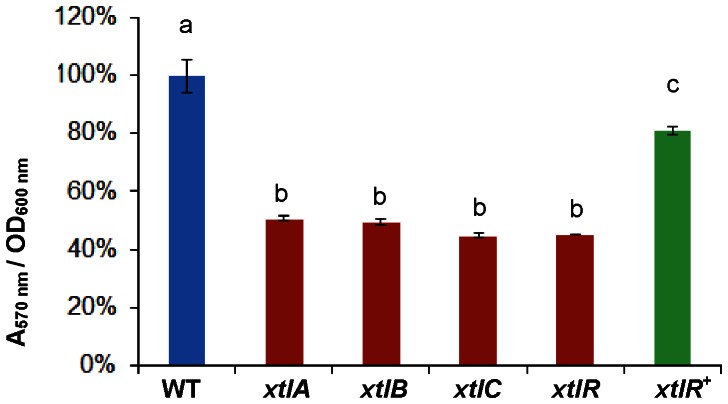
Biofilm formation by *P.putida* BW11M1 and xantholysin-deficient mutants. Abbreviations as in Fig. 4. Error bars indicate standard deviations. The analysis of variance (ANOVA) test was used to evaluate significant differences (p<0.001; indicated with different letters above the bars) between the wild type (set to 100%; blue), mutants (red), and complemented regulatory mutant (green).

Compared to wild-type BW11M1 cells, the mutants deficient in anti-*Xanthomonas* activity showed significantly reduced biofilm formation at 30°C, but still retained almost half of the wild-type capacity (Fig. 4F, Fig. 5). This indicates that xantholysin contributes to BW11M1 biofilm formation under these experimental conditions, acting together with other factors. Biofilm formation by the complemented regulatory mutant was increased to about 80% of wild-type biofilm formation (Fig. 5).

The lack of full complementation of the regulatory mutant to wild-type level of biofilm formation, also observed to a certain extent for antagonistic and hemolytic phenotypes, may be due to some polar effect of the *xtlR* insertion on expression of the downstream *oprM*-like gene *xtlF*, encoding the presumed outer membrane component of the tripartite transport system, possibly diminishing xantholysin export. A complementation construct carrying both *xtlR* and *xtlF* could not be obtained to test this hypothesis.

### Xantholysin A: a Novel Cyclic Lipotetradecadepsipeptide

LC-MS analysis of semi-purified extracts revealed the presence of four lipopeptide compounds with molecular masses 1775.4 Da (main compound, xantholysin A), 1761.2 Da (minor variant 1, xantholysin B), 1802.0 Da (minor variant 2, xantholysin C) and 1775.2 Da (minor unidentified xantholysin variant 3, xantholysin D) (Fig. S6, Fig. S7). Only xantholysin A could be successfully isolated in sufficient quantity to allow adequate NMR analysis (Fig. S8, Table S6).

The 2D ^1^H-^1^H TOCSY spectrum of xantholysin A in DMF-d7 (Fig. S9) allowed the identification of residues corresponding to five Leu, one Ile, two Val, five Glx and one Ser by means of their characteristic amino acid correlation patterns. The presence of a 3-hydroxydecanoic acid (HDA) moiety was determined from combined analysis of the ^1^H-^1^H TOCSY and ^1^H-^13^C HSQC spectra and comparison with the chemical shifts reported for pseudodesmin A in dimethylformamide solution [17], [60]. The sequence (HDA-Leu1-Glx2-Glx3-Val4-Leu5-Glx6-Ser7-Val8-Leu9-Glx10-Leu11-Leu12-Glx13-Ile14) was established by observing sequential H^α^-H^N^ cross-peaks in the 2D ^1^H-^1^H NOESY spectrum (Fig. S10). The TOCSY and NOESY spectra also revealed the presence of four individual pairs of mutually correlated amide resonances originating from primary amide functional groups. Correlations in the NOESY spectrum between these resonances and the CH_2_^γ^ and CH_2_^β^ resonances of the Glx residues allowed specific assignment of each amide resonance pair to a Glx residue, effectively establishing the identity of residues 3, 6, 10 and 13 to be Gln. The absence of any such correlations to the CH_2_^γ^ and CH_2_^β^ resonances of residue 2 and any further primary amide group resonance pair in the spectrum established the identity of residue 2 as Glu. The ^1^H-^13^C HMBC spectrum allowed the assignment of several ^13^C carbonyl resonances from the ^2^J_CH_ coupling with the H^N^ resonances (Table S6). The resulting peptide sequence (Leu-Glu-Gln-Val-Leu-Gln-Ser-Val-Leu-Gln-Leu-Leu-Gln-Ile; Fig. 2) largely confirmed the phylogeny-based prediction using an organism-specific subset of experimentally confirmed A-domain sequences. A high-resolution mass spectrum confirmed the chemical formula as C_84_H_146_N_18_O_23_ (exact mass: 1775.0764 Da; Fig. S11), thus indirectly providing additional support for the number of Gln and Glu residues and the length (C_10_) of the fatty acid chain.

At this point, the only feature of the proposed xantholysin A structure which still remained to be confirmed was the depsi bond between Ile14 and Ser7. The predicted molecular mass of the above sequence when considering it as a linear lipopeptide deviates 18 Da with the experimental value, in agreement with the expected presence of the lactone bond. Based on previous experience in assigning cyclic lipodepsipeptides [60], the only direct demonstration of the existence of this bond would be by detecting a ^3^J_CH_ correlation between the C-terminal Ile14 carbonyl and the Ser7 CH_2_^β^ resonances in the HMBC spectrum. While spectral overlap prevented convincing observation of such correlation, indirect but clear evidence of the presence of the depsi bond is found in the chemical shift of the Ser7 CH_2_^β^ protons, which is significantly higher (4.38 ppm and 4.58 ppm) compared to their random coil values (3.79 ppm and 3.95 ppm) [61]. A similar downfield chemical shift for protons neighboring the depsi bond is observable for other cyclic lipodepsipeptides as well [60] and is explained by an anisotropic deshielding effect created by the carbonyl double bond. This phenomenon can thus be seen as a characteristic feature for this class of compounds, allowing quick recognition of the presence and location of the depsi bond. Importantly, the only other chemically feasible location of the depsi bond – the alcohol group of the HDA moiety – can in this way be excluded, since the HDA H^β^ resonance does not experience such a strong downfield shift in comparison to other cyclic lipodepsipeptides that were assigned by NMR [17], [60].

The modest quantities obtained for the minor xantholysin variants did not allow to record NMR data of sufficient quality for a *de novo* analysis and full spectral assignment (Fig. S12, Fig. S13). Nevertheless, direct comparison of the ^1^H-^1^H TOCSY and ^1^H-^13^C HSQC spectra for xantholysin B and C with the corresponding spectra measured on xantholysin A allowed to identify the nature of the variation. For both compounds, most of the ^1^H and ^13^C chemical shifts were nearly identical to xantholysin A, demonstrating their overall high similarity. In the case of xantholysin B, the single observed difference is a substitution of the Ile14 spin system for a Val spin system, explaining the 14 Da mass difference (Fig. S14). The spectra indicated the lipid tail moiety of xantholysin C to consist of 3-hydroxydodec-5-enoate (Fig. S13), accounting for the 26 Da mass increase. High-resolution mass spectra of both compounds confirmed the chemical formulae to be C_83_H_144_N_18_O_23_ (exact mass: 1761.0574 Da) and C_86_H_148_N_18_O_23_ (exact mass: 1801.0900 Da) for xantholysin B and C, respectively (Fig. S15, Fig. S16). Considerations of the ^13^C chemical shifts within the lipid tail moiety of xantholysin C suggest the double bond to be in the *cis* configuration (Fig. S17). This is reminiscent of the promiscuous lipoinitiatory activity reported for *Pseudomonas corrugata* producing similar amounts of corpeptin A (HDA) and corpeptin B (3-hydroxydodec-5-enoic acid) [62]. In most lipopeptide-producing *Pseudomonas*, promiscuity is observed with respect to chain length of the incorporated saturated 3-hydroxy fatty acids. Relaxed amino acid substrate specificity, as observed here for the terminal module in XtlC giving rise to minor amounts of xantholysin B, is common among *Pseudomonas* NRPS systems [5], [8], [9], [43].

Xantholysin-like lipopeptides with antiviral activity were isolated from *Pseudomonas* sp. RtIB026. The main compound MA026 (PubChem CID 10285742) differs by two consecutive amino acids (Leu10-Gln11 in MA026 versus Gln10-Leu11 in xantholysin A). Congener R1MA026 (with Val9-Leu14) contains an additional switch of two residues compared to xantholysin B (with Leu9-Val14). Congener R2MA026 differs from MA026 by a 3-hydroxydodec-5-enoyl instead of a 3-hydroxydecanoyl fatty acid moiety (same as the difference between xantholysins A and C). Given these striking similarities, it would be of interest to identify the biosynthetic genes of strain RtIB026 (patent strain FERM BP-7436) to compare the modular structure of its NRPSs with those of the xantholysin system. The MA026 structure suggests a peculiar A-domain switch between the last module of the second enzyme and the first module of the third enzyme in RtIB026, compared to XtlB and XtlC, respectively. Given the phylogenetic separation of the Val- and Leu-selective A-domains of module 9 and 14 in xantholysin (Fig. 3), the biosynthesis of an as yet unidentified Val9-Leu14 congener by the xantholysin synthetases is unlikely. Since for none of these RtIB026 structures a detailed NMR analysis is available that would allow comparison to the xantholysins, it is at this point still unclear whether indeed such striking biosynthetic variation is occurring or whether the RtIB026 structures require revision.

## General Conclusions

### Distinct Antibiotic Properties of Xantholysin

The identification of xantholysin adds to the growing list of cyclic lipopeptides produced by pseudomonads. Like several other *Pseudomonas* lipopeptides [3], xantholysin appears to promote surface colonization of its producer, *P. putida* BW11M1, a strain isolated from the rhizosphere of banana plants. However, unlike most pseudomonad lipopeptides, antimicrobial activity is not confined to fungi and Gram-positive bacteria, but also extends to some Gram-negative strains, including several xanthomonads. Members of this γ-proteobacterial genus that are colonizing similar plant niches as pseudomonads [29]–[32], are also susceptible to WLIP, a member of the viscosin group [17], but *Xanthomonas* strains exhibit differences in sensitivity to both lipopeptides. During this study it became evident that, in addition to xantholysin, strain BW11M1 produces one or more as yet unidentified antimicrobial factor(s), also affecting a number of xanthomonads. While xantholysin does not exert a growth-inhibitory effect on fellow pseudomonads, *P. putida* BW11M1 also produces a plant lectin-like bacteriocin to target other pseudomonads, including members of the *P. syringae* group [35], [63], indicating that both ribosomally and non-ribosomally synthesized antimicrobials are part of its armory deployed against competitors. Antagonism among plant-associated γ-proteobacterial relatives has been described for some other types of pseudomonad secondary metabolites: the modified amino acid 3-methylarginine causing inhibition between *P. syringae* pathovars [64]; the salicylate-containing *P. putida* antibiotic promysalin, targeting other pseudomonads [65]; oxyvinylglycines active against *Erwinia amylovora* [66], [67]. In view of the structural similarity of xantholysins to certain pseudomonad lipopeptides with antiviral activity (viz. MA026 and its congeners), future experiments should also address the effects of xantholysins on enveloped viruses. Antiviral activity has been reported for some other lipopeptides, such as viscosin and surfactin [3].

### Xantholysin and Entolysin Synthetases: Similar Enzymes but Very Different Products

**Figure 6:**
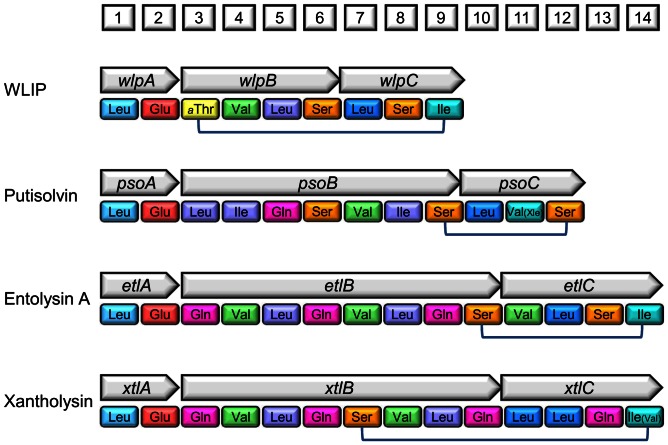
Modular architecture of the xantholysin synthetases and other *Pseudomonas* enzymes with similar A domains. The respective starter NRPS genes are located distantly from the gene pairs encoding the middle and terminating NRPSs, except for the putisolvin operon *psoABC* [16]. Synthesis of the peptide moiety by the consecutive action of the modules (with only A domains shown) proceeds in a co-linear fashion, with incorporation of the respective amino acid at the positions corresponding to the numbered boxes. If a minor lipopeptide variant with a different amino acid at a certain position has been identified, this residue is shown in parentheses with smaller font. Xle indicates that the residue’s identity (either Leu or Ile) was not resolved in putisolvin II. The connected residues form a depsi bond. Similarity between the different systems is visualized according to Rokni-Zadeh et al. [18]: two domains with a patristic distance <0.45 (summed branch lengths in a maximum-likelihood tree constructed from aligned A-domain sequences; see Fig. 3) are represented in the same color.

The genetic backbone of the xantholysin NRPS system bears considerable similarity to the one for entolysin biosynthesis by the *P. putida* relative *P. entomophila* L48 [10], both consisting of a fourteen-unit assembly line with three enzymes operating in co-linear mode (Fig. 6). Conservation of modular architecture is most striking for the initiatory enzymes XtlA and EtlA, both composed of two modules, and the subsequently acting enzymes, XtlB and EtlB. Overall, the latter enzymes share eight probably equifunctional modules but a single differently positioned serine-incorporating unit generates a different amino acid sequence stretch. Furthermore, this apparent “serine switch” also shifts the site of macrocyclization with three residues, such that xantholysin carries an octacyclic moiety instead of the pentacyclic structure in entolysin. Although the three NRPS enzymes from strains BW11M1 and L48 share relatively high pairwise sequence homology (70.5% amino acid identity for ∼15,440 concatenated residues), the architectural divergence engenders synthesis of quite different molecules that can be assigned to separate groups within the *Pseudomonas* lipopeptide classification, based on amino acid sequence (length and similarity) and presence/type of macrocycle [4]–[6].

### Unexpected Similarities among Pseudomonas NRPS Systems: the Advantage of being Modular

Phylogenetic comparison of the respective NRPS domains, the linked LuxR-family regulator and the associated tripartite export system consistently points to evolutionary relatedness of the components of the xantholysin and entolysin systems with those of *P. putida* strains generating lipopeptides that – at first glance – show little structural similarity: putisolvin (twelve amino acids, tetracyclic; [16]) and WLIP (nine amino acids, heptacyclic; [17]). These four *P. putida* lipopeptides share a very similar A-domain pair in the initiatory synthetases (EtlA, PsoA, WlpA, XtlA; Fig. 6). The equivalent modules in the *P. fluorescens* starter enzymes for incorporation of the same dipeptide (Leu-Glu) in viscosin-group lipopeptides cluster separately from the *P. putida* ones [18]. From Fig. 6 it is also apparent that xantholysin synthetase B shares a core of five phylogenetically related contiguous modules with putisolvin synthetase B (A-domains for Leu/Ile-Gln-Ser-Val-Leu/Ile). Comparison of the A-module architecture of entolysin, WLIP and putisolvin synthetases C points to a tripartite terminating block composed of A-domains with common evolutionary origin, but arranged in a different order in PsoC compared to EtlC and WlpC.

Collectively, these observations suggest that these pseudomonads probably have recruited and reshuffled gene modules to engineer novel NRPS assembly lines for synthesis of new structural lipopeptide variants. In combination with rapidly accumulating genomic sequence data, high-throughput exploration of this part of the “parvome” [68] becomes feasible with recently developed strategies of mass spectral metabolic profiling of live microbial colonies [44], [69]. In addition to identification of new molecules and associated biological activities, this will likely provide insight in the extent of modular diversity among these biosynthetic enzymes, and how this has been generated and further evolved in prominent lipopeptide producers such as *Pseudomonas* and *Bacillus*.

## Materials and Methods

### Bacterial Strains, Plasmids and Culture Conditions

Bacterial strains and plasmids used are described in Table 1 and Table S4. TSB growth medium (trypticase soy broth, 30 g/L; BD Biosciences) was used for *Pseudomonas* and *Xanthomonas* strains at 30°C and for *Klebsiella pneumoniae* ATCC 13883 and *Staphylococcus aureus* ATCC 6358 at 37°C. *Aeromonas hydrophila* ATCC 7966, *Agrobacterium tumefaciens* A208, *Azospirillum brasilense* Sp7, *Burkholderia vietnamiensis* LMG 10927, *Mycobacterium smegmatis* DSM 43756, *Rhodococcus erythropolis* N11, *Serratia entomophila* DSM 12358, *Sphingomonas wittichii* RW1, *Variovorax paradoxus* LMG 1797 and *Yersinia enterocolitica* LMG 7899 were grown at 30°C, and *Bordetella avium* 197N, *E. coli* and *Salmonella enteritidis* ATCC 13076 at 37°C, in LB (tryptone 10 g/L, yeast extract 5 g/L, NaCl 10 g/L). Nutrient broth (8 g/L) was prepared for growing *Bacillus megaterium* ATCC 13632 and *Erwinia amylovora* CFBP 1430 (at 30°C), and for *Citrobacter freundii* ATCC 8090, *Enterobacter aerogenes* ATCC 13048, *Proteus vulgaris* LMM 2011 and *Shigella flexneri* LMG 10472 (at 37°C). YEP (yeast extract 10 g/L, bacterial peptone 10 g/L) was used for *Burkholderia cepacia* LMG 1222, TY (tryptone 5 g/L, yeast extract 3 g/L, CaCl_2_ 7 mM) for *Bacillus subtilis* LMG 7135 (all grown at 30°C), and MRS (Difco lactobacilli MRS broth 55 g/L) for *Lactobacillus plantarum* LMG-P21295 (cultured at 30°C) and *Lactobacillus rhamnosus* GG (LMG 6400, at 37°C). *Streptomyces coelicolor* A3(2) and *Streptomyces lividans* TK24 were cultivated in a medium composed of 10 g casitone, 5 g yeast extract and 5 g glucose per liter at 30°C. For solid media 15 g per liter agar was added. Antibiotics were added at the following concentration when required: kanamycin (50 µg/ml), ampicilin (100 µg/ml), tetracycline (20 µg/ml).

**Table 1:** Bacterial strains and plasmids.

Strain or plasmid	Description and characteristics^a^	Source or Reference
**Strains**		
*E. coli* TOP10F’	F′*lac*Iq, Tn*10*(TetR) *mcr*A (*mrr*-*hsd*RMS-*mcr*BC) 80*lac*ZM15 *lac*X74 *rec*A1 *ara*D139 (*ara leu*) 7697 *gal*U *gal*K *rps*L (StrR) *end*A1 *nup*G	Invitrogen
*E. coli* EPI300-T1^R^	Host strain for pCC2FOS derivatives	Epicentre Biotechnologies
*E. coli* DH5α	*supE44* Δ*lacU169* (*Φ80 lacZ*Δ*M15*) *hsdR17 recA1 endA1 gyrA96 thi-1 relA1*	[70]
*E. coli* HB101 (pRK2013)	Helper strain in triparental conjugation, Km^r^	[71]
*P. putida* BW11M1	Banana rhizosphere (Sri Lanka)	[33]
*P. putida* CMPG2183	BW11M1 *xtlA* mutant	This study
*P. putida* CMPG2187	BW11M1 *xtlB* mutant	This study
*P. putida* CMPG2198	BW11M1 *xtlC* mutant	This study
*P. putida* CMPG2201	BW11M1 *xtlR* mutant	This study
*X. alfalfae* subsp. *alfalfae* LMG 497	Pathovar reference strain; *Medicago sativa* (Sudan)	BCCM^b^
*X. axonopodis* pv. *manihotis* LMG 784	Pathovar reference strain; *Manihot esculenta* (Brazil)	BCCM
*X. campestris* pv. *campestris* LMG 582	*Brassica* sp. (Belgium)	BCCM
*X. citri* pv. *malvacearum* LMG 761	Pathovar reference strain; *Gossypium* sp. (Sudan)	BCCM
*X. hortorum* pv. *hederae* LMG 7411	*Hedera helix* (USA)	BCCM
*X. sacchari* LMG 471	Type strain; *Saccharum officinarum* (Guadeloupe)	BCCM
*X.* sp. pv. *zinniae* LMG 8692	Pathovar reference strain; *Zinnia elegans* (Australia)	BCCM
*X. translucens* LMG 12921	*Anthurium andreanum* (USA)	BCCM
*X. translucens* pv. *cerealis* LMG 679	Pathovar reference strain; *Bromus inermis* (USA)	BCCM
*X. translucens* pv. *graminis* LMG 726	Pathovar reference strain; *Dactylis glomerata* (Switzerland)	BCCM
*X. translucens* pv. *hordei* LMG 737	Pathovar reference strain; *Hordeum vulgare* (India)	BCCM
*X. vasicola* pv. *holcicola* LMG 736	Pathovar reference strain; *Sorghum bicolor* (New Zealand)	BCCM
*X. vasicola* pv. *musacearum* LMG 785	Pathovar reference strain; *Ensete ventricosum* (Ethiopia)	BCCM
*X. vasicola* pv. *musacearum* LMG 7431	*Musa* sp. (Ethiopia)	BCCM
*X. oryzae* pv. *oryzae* PXO99	Race 6; *Oryza sativa* (The Philippines)	IRRI^c^
*X. oryzae* pv. *oryzae* PXO112	Race 5; *Oryza sativa* (The Philippines)	IRRI
*X. oryzae* pv. *oryzae* PXO340	Race 3; *Oryza sativa* (The Philippines)	IRRI
**Plasmids**		
pCC2FOS	Copy control fosmid	Epicentre Biotechnologies
pJB3Tc20	Broad-host-range cloning vector, Ap^r^, Tc^r^	[72]
pCMPG6126	Fosmid clone containing *xtlA*	This study
pCMPG6127	Fosmid clone 12H4 containing *xtlB* and partial *xtlC*	This study
pCMPG6128	Fosmid clone 19F2 containing *xtlC* and partial *xtlB*	This study
pCMPG6204	pJB3Tc20 containing *xtlR*, Tc^r^	This study

Other bacteria used as indicator strains are listed in Table S4.

a Antibiotic resistance phenotypes: Ap^r^, ampicillin resistance, Km^r^, kanamycin resistance; Tc^r^, tetracycline resistance.

b BCCM, Belgian Coordinated Collections of Microorganisms.

c IRRI, International Rice Research Institute.

### Antibacterial Activity Assays by Agar Diffusion

*P. putida* BW11M1 was grown in 5 ml TSB for 8 h with shaking (200 rpm). Two µl of this culture (OD_600_ ∼1.8) was spotted onto a TSB agar and incubated overnight at 30°C. After exposure to chloroform vapor for 30 min to kill producer bacteria, the plates were opened in a laminar flow chamber to evaporate residual chloroform during another 30 min. Three ml of molten, semisolid agar (0.5%) was inoculated with 100 µl of a bacterial indicator cell culture (∼10^8^–10^9^ CFU/ml) and poured onto the plates as an overlay. Following day, plates (three replicates) were evaluated for growth inhibition halos in a turbid lawn of indicator cells. Protease sensitivity of the antimicrobial activity was verified by spotting 10 µl of proteinase K (20 mg/ml) near a chloroform-killed producer colony prior to overlay.

### Growth Inhibition of Fungi

An agar plug with fungal mycelium, grown for 1–2 weeks on half-strength PDA (potato dextrose agar) medium was placed at the center of an agar plate with appropriate medium at 20°C. PTA medium (potato dextrose broth 12 g, TSB 3 g, agar 15 g per liter) plates were used for most fungi. *Alternaria porri* was grown on 6CA-TSB medium (Bambix 6 cereals 10 g, TSB 15 g, agar 15 g per liter). 5 µl of overnight cell culture of *P. putida* BW11M1 and selected xantholysin-deficient mutants were spotted at a distance of ∼3 cm from the inoculum plug and incubated at 20°C for several days up to two weeks, depending on the growth rate of the fungi, before assessing the occurrence of mycelial growth inhibition zones. Indicator fungi are listed in Table S5.

The effect of xantholysin production on *Agaricus bisporus* was carried out as described by Bessette [52] with minor modifications. Briefly, 20 µl of an overnight *Pseudomonas* culture (∼10^8^–10^9^ CFU/ml) was spotted onto horizontally sliced pilei of mushroom basidiocarps (*Agaricus bisporus*). After absorption of the liquid by the sterile surface, mushrooms were incubated overnight at 30°C, sealed in a closed plastic box and saturated with water to prevent drying out. Following day, mushrooms were scored for the presence of browning. Cultures of wild type and mutants were tested in parallel (three independent biological repeats).

### Isolation and Characterization of Mutants with Abolished Anti-Xanthomonas Activity

A BW11M1 plasposon pTn*Mod*-OKm’ mutant library [35] was screened for mutants with abolished activity against indicator strain *X. alfalfae* subsp. *alfalfae* LMG 497 by the agar diffusion method. Each mutant from a 96-well microtiter plate was stamped onto TSB agar square petri dishes (12×12 cm) with a 96-prones replicator. Mutants lacking anti-LMG 497 activity were selected and confirmed twice. The plasposon-interrupted genes were identified by the plasmid rescue method [36] and subsequent sequencing of the flanking regions using pTn*Mod*-OKm’-specific primers PGPRB-0293 (5′-TCTGGCTGGATGATGGGGCG-3′) and PGPRB-0294 (5′-CGGTTCCTGGCCTTTTGCTGGC -3′).

### Fosmid Library Construction and Screening

For construction of a *P. putida* BW11M1 genomic library in fosmid pCC2FOS, the CopyControl HTP Fosmid Library Production Kit protocol (Epicentre Biotechnologies) was applied. Based on the flanking sequences identified in the plasposon mutants, pairs of primers were designed to screen for fosmid clones containing the corresponding genomic DNA regions by colony PCR. Selected clones were compared by end-sequencing using pCC2FOS-specific primers PGPRB-1319 (5′-GTACAACGACACCTAGAC-3′) and PGPRB-1320 (5′-CAGGAAACAGCCTAGGAA-3′). Finally, three fosmids with DNA fragments covering the genomic regions of interest were sequenced at Macrogen (Korea). The insert size of the fosmids was estimated by separation of *Not*I-generated fragments using pulsed-field gel electrophoresis. A high copy number of the fosmids was induced by treating cells with the CopyControl Induction solution, prior to extraction of the fosmids with the FosmidMAX DNA purification kit (Epicentre Biotechnologies). The contigs with the regions containing the functionally characterized genes were submitted to GenBank (accession numbers KC297505 and KC297506).

### Bioinformatic Analyses

Domains were identified using NRPS analysis tools [73], [74]. The NRPSpredictor2 tool was used to predict amino acid specificity of A domains [38]. Amino acid sequences were aligned with MUSCLE for maximum-likelihood tree construction using (PhyML with JTT matrix; [75]) as implemented in Geneious Pro (version 5.6.3).

### Complementation of xtlR Regulatory Mutant

Complementation of mutant CMPG2201 was performed as previously described [17]. Briefly, the *xtlR* gene was amplified using Platinum *Pfx* DNA polymerase (Invitrogen) with primers PGPRB-8013 (5′-TTGCTCTAGATCAGGTGCCGGCCATCCAGC-3′) and PGPRB-8014 (5′-CTCGGAATTCGGATCAAGGAAGACGACGAC-3′) and cloned into the pJB3Tc20 vector using the *Xba*I and *Eco*RI sites. The resulting construct (pCMPG6204) was transferred to *E. coli* DH5α. Conjugative transfer of pCMPG6204 to *P. putida* BW11M1 mutant CMPG2201 was achieved by triparental conjugation using *E. coli* HB101 bearing pRK2013 as helper strain. The phenotypes of a selected transconjugant were compared to the regulatory mutant.

### Swarming Motility, Biofilm Formation and Hemolytic Activity

Swarming ability was studied by spotting 2 µl of 8 h-grown cells on TSB medium solidified with 0.8% agar and evaluating surface spreading after incubation at 30°C for 16 to 24 h (three repeats for each sample). Biofilm formation was assayed according to Janssens et al. [76] by using flat-bottom 96-well microtiter plates and polystryrene pegs (Nunc, Sanbio b.v.). Cells grown for 8 hours were adjusted to OD_600_ ∼1.0 and then diluted 100-fold as initial culture (200 µl per well, 8 wells per strain). After 2 days of incubation at 30°C, biofilm formation was visualized by staining pegs with 0.1% crystal violet. Stained biofilms were subsequently extracted with 30% acetic acid and quantified by measuring absorbance of the solution at 570 nm. Hemolytic activity was assayed by spotting 2 µl of overnight-grown cells on TSB agar with 5% horse blood (Oxoid) plates and hemolysis halos were examined after incubating plates overnight at 30°C.

### Lipopeptide Extraction and Purification

Overnight TSB-grown BW11M1 cell culture was used to inoculate fresh TSB medium at a volume ratio of 1∶100 and incubated at 30°C for at least 2 days with shaking (200 rpm). After removing cells through centrifugation (6370 *g*, 30 min, 4°C), the supernatant was acidified by adding 9% HCl to lower the pH to pH 2–3. The precipitate formed was isolated by centrifugation (6370 *g*, 30 min, 4°C) and washed twice with acidified distilled water (pH 3). Methanol was used to dissolve the pellet and the solution was kept at 4°C till further purification. After evaporation of solvent, the slurry was extracted with cold methyl *tert*-butyl ether to precipitate the peptide fraction. This procedure was repeated three times to obtain a crude peptide-rich mixture which was further purified by preparative RP-HPLC. Samples were dissolved in methanol (50 mg/ml) and eight injections of 1 ml were performed on a Phenomenex column (Luna C18(2) 250×21.2 mm, 5 µm particle size) operating at a flow rate of 17.5 ml/min. The ratio of the mobile phase solvents, 5 mM ammonium acetate in water and acetonitrile, was changed in a linear fashion from 50∶50 to 10∶90 over a time span of 15 minutes. The major compound (xantholysin A) was collected based on UV detection at λ = 214 nm. Two additional minor lipopeptides, shown to be xantholysin variants B and C, were isolated by analytical HPLC using an Agilent 1100 series instrument equipped with a Phenomenex column (Luna C18(2), 250×4.6 mm, 5 µm particle size) and operating at a flow rate of 0.8 ml/min. The ratio of mobile phase solvents, 5 mM ammonium acetate in water and acetonitrile, was changed from 35∶65 to 25∶75 over a time span of 15 minutes. To identify bioactive fractions, samples from collected fractions were spotted on a TSB agar plate and overlayed with indicator cells after evaporation of the solvent (with non-polar phase solvent as negative control).

### Xantholysin Structure Elucidation

Initial mass spectra were acquired on a quadrupole orthogonal acceleration time-of-flight mass spectrometer (Synapt G2 HDMS, Waters, Milford, MA). Samples were infused at 3 µl/min and spectra were obtained in positive ionization mode with a resolution of 15000 FWHM and accuracy of 0.5 ppm RSD using leucine enkephalin as lock mass. Additional LC-MS data were collected on the extracted mixture prior to purification with an Agilent 1100 Series HPLC with an ESI detector type VL, equipped with a Phenomenex column (Luna C18(2), 250×4.60 mm, 5 µm particle size). The flow rate was 1 ml/min. The mobile phase solvents, 5 mM ammonium acetate in water and acetonitrile, were linearly changed from a 25∶75 ratio to a 0∶100 ratio over a time span of 15 minutes.

High-resolution mass spectra were recorded on an Agilent 6220A time-of-flight mass spectrometer, equipped with an Agilent ESI/APCI multimode source. The ionization mode was set to APCI (atmospheric pressure chemical ionization), while the mass spectra were acquired in 4 GHz high-resolution mode with a mass range set to 3200 Da. All NMR measurements on xantholysin A were performed on a sample of 6.5 mg dissolved in 600 µl DMF-*d7* (Eurisotop). High quality HP-7 (New Era Ent. Inc) NMR tubes were used. NMR experiments were performed on a Bruker Avance III spectrometer operating at 500.13 MHz and 125.76 MHz for ^1^H and ^13^C, respectively, and equipped with a 5 mm ^1^H BBI-Z probe. Due to the low absolute quantities of compound after purification, the xantholysin variants were dissolved in 10 µl DMF-*d7* for measurements using 1 mm capillaries. In this case, NMR experiments were performed on a Bruker Avance II spectrometer operating at 700.13 MHz and 176.04 MHz for ^1^H and ^13^C, respectively, and equipped with a 1 mm ^1^H,^ 13^C,^ 15^N TXI-Z probe. For both analysis of xantholysin A and its variants, sample temperature was set to 55°C throughout, as significant line broadening effects occurred at lower temperatures. 2D spectra measured for structure elucidation include a ^1^H-^1^H TOCSY with a 90 ms MLEV-17 spinlock, a sensitivity-improved, multiplicity edited, ^1^H-^13^C gHSQC applying adiabatic 180° pulses, a ^1^H -^1^H NOESY with a 300 ms mixing time and a ^1^H-^13^C gHMBC experiment optimized for a carbon-proton coupling of 8 Hz. Standard pulse sequences as present in the Bruker library were used throughout. Typically, 2048 data points were sampled in the direct dimension for 256 data points in the indirect one, with the spectral width respectively set to 12 ppm along the ^1^H dimension and 110 ppm (gHSQC) or 220 ppm (gHMBC) along the ^13^C dimension. For 2D processing, the spectra were zero filled to obtain a 2048×2048 real data matrix. Before Fourier transformation, all spectra were multiplied with a squared cosine bell function in both dimensions except for the gHMBC where a squared sine bell was applied.

## Supporting Information

Figure S1 **Cladogram representation of a maximum-likelihood** **tree inferred from amino acid sequence alignment of condensation domains extracted from characterized** ***Pseudomonas*** **NRPSs.** The lipopeptide-specific codes are specified in Fig. 3, with the xantholysin-related ones in bold. Clusters are differentiated according to the type of C-domain by color: conventional domains (green), dual epimerization/condensation domains (blue), and N-acylating starter domains (red). The tree was rooted with the divergent C1 domain of SyrE (in black).
(PDF)

Figure S2 **Maximum-likelihood** **tree of thioesterase domains of characterized LP-producing** ***Pseudomonas*** **NRPSs.** The clusters with tandemly organized domains TE1 (red) and TE2 (green) are shown in different colors. Lipopeptide-specific codes as in Fig. 3, with the xantholysin-related ones in bold. The tandem-TE domains of lysobactin synthetase LybB from *Lysobacter* sp. ATCC 53042 and the mono-TE domain of syringomycin synthetase SyrE from *P. syringae* pv. *syringae* B301D are included for comparison. The internal residue involved in cyclization with the carboxyterminal amino acid is specified for each TE1/TE domain (NA = not applicable for the linear syringafactins; OH-Phe = β-hydroxy phenylalanine). The scale bar represents 0.5 substitutions per site.
(PDF)

Figure S3 **Comparison of cognate regulators and associated export systems in lipopeptide-synthesizing** ***Pseudomonas*** **.** Maximum-likelihood trees inferred from amino acid sequence alignments of (A) LuxR-family regulators encoded by genes linked with the respective initiatory NRPS genes and (B) the components of tripartite MacA/MacB/OprM-like export systems (concatenated sequences; same phylogeny was obtained for the three individual sequence sets (data not shown)). Lipopeptide-specific codes as in Fig. 3, with the xantholysin-related ones in bold. The regulatory gene encoding SyrF (panel A) is located downstream of *syrE*. In panel B, the Syf and Arf systems are not included (no sequences available for the corresponding OprM-like protein). The scale bars represent 0.09 (panel A) and 0.04 (panel B) substitutions per site. The xantholysin cluster (blue) and viscosin cluster (green) are shown in different colors.
(PDF)

Figure S4 **Growth inhibition of representative indicator bacteria by purified xantholysin.** 10 µl-samples of purified xantholysin in methanol, containing 18 µg (A) or 9 µg (B) were spotted on an agar plate and overlaid with indicator cells after solvent evaporation. Methanol control spots did not cause growth inhibition.
(PDF)

Figure S5 **Antifungal activity of xantholysin.** Growth inhibition of fungi by *P. putida* BW11M1 (WT) and xantholysin-deficient mutants (*xtlA*, *xtlB*, *xtlC*, *xtlR;* CMPG2183, CMPG2187, CMPG2198, and CMPG2201, respectively). The application pattern of bacterial cell suspensions spotted around a centrally positioned plug with fungal mycelium is shown schematically in (A). (B) *Alternaria longipes* CBS 620.83, (C) *Ascochyta pisi* MUCL30164, (D) *Botrytis cinerea* MUCL30158, (E) *Colletotrichum gloeosporoides* SR24, (F) *Fusarium culmorum* MUCL30162, (G) *Fusarium graminearum* MUCL30161, (H) *Fusarium oxysporum* MUCL909, (I) *Fusarium oxysporum* MUCL30160, (J) *Fusarium oxysporum* f. sp. *radices-lycopersi* CBS 873.95, (K) *Fusarium oxysporum* f. sp. *vasinfectum* CBS 410.90, (L) *Gloeosporium musarum* MUCL500, (M) *Gloesporium solani* CBS 194.32, (N) *Nectria haematococca* MUCL20259, (O) *Pyrenophora tritici-repentis* MUCL30217, (P) *Rhizoctonia solani* CBS 211.84.
(PDF)

Figure S6 **Chromatographic separation of xantholysin congeners.** LC-MS chromatogram of the extracted mixture prior to purification using UV-detection at 214 nm.
(PDF)

Figure S7 **Mass spectrometry of xantholysin congeners.** Mass spectra of xantholysin A and its variants obtained during LC-MS analysis of the extracted mixture prior to purification. Preliminary experiments with a high-resolution instrument showed that the peaks represent ions with charge z = 2.
(PDF)

Figure S8 **1D NMR analysis of xantholysin A.** 1D ^1^H spectrum of xantholysin A in DMF-d7 solution, 55°C, 500 MHz.
(PDF)

Figure S9 **2D NMR analysis of xantholysin A.** 2D ^1^H-^1^H TOCSY spectrum of xantholysin A in DMF-d7 solution, 55°C, 500 MHz, revealing the characteristic amino acid spin system patterns.
(PDF)

Figure S10 **Nuclear Overhauser effect spectroscopy of xantholysin A.** Established NOE contacts in xantholysin A, observed in a 2D ^1^H-^1^H NOESY spectrum with 300 ms mixing time.
(PDF)

Figure S11 **High resolution mass spectrum of xantholysin** A. (A) Full mass spectrum. (B) Zoom on the [M+H]^+^ molecular ion peaks. Expected exact mass of xantholysin A (C_84_H_146_N_18_O_23_)+H^+^: 1776.0881 Da; observed exact mass of xantholysin A+H^+^: 1776.0837 Da.
(PDF)

Figure S12 **1D NMR analysis of xantholysin B.** 1D ^1^H spectrum of xantholysin variant 1, in DMF-d7 solution, 55°C, 700 MHz.
(PDF)

Figure S13 **1D NMR analysis of xantholysin C.** 1D ^1^H spectrum of xantholysin variant 2, in DMF-d7 solution, 55°C, 700 MHz. The inset shows the signals arising from the alkene protons of the 3-hydroxydodec-5-enoic acid moiety, on top of an unidentified resonance from an impurity.
(PDF)

Figure S14 **2D NMR analysis of xantholysin B.** 2D ^1^H-^1^H TOCSY spectrum of xantholysin variant 1, in DMF-d7 solution, 55°C, 700 MHz, revealing the characteristic amino acid spin system patterns.
(PDF)

Figure S15 **High resolution mass spectrum of xantholysin B.** (A) Full mass spectrum. (B) Zoom on the [M+H]^+^ and [M+Na]^+^ molecular ion peaks. Expected exact mass of xantholysin B (C_83_H_144_N_18_O_23_)+H^+^: 1762.0724 Da; observed exact mass of xantholysin B+H^+^: 1762.0647 Da.
(PDF)

Figure S16 **High resolution mass spectrum of xantholysin C.** (A) Full mass spectrum. (B) Zoom on the [M+H]^+^ and [M+Na]^+^ molecular ion peaks. Expected exact mass of xantholysin C (C_86_H_148_N_18_O_23_)+H^+^: 1802.1037 Da; observed exact mass of xantholysin C+H^+^: 1802.0973 Da.
(PDF)

Figure S17 **NMR analysis of fatty acid moiety in xantholysin C.** ^1^H and ^13^C chemical shift comparison between the lipid tail of xantholysin A and variant 2. (A) Experimental ^1^H (red) and ^13^C (blue) chemical shifts of the xantholysin 3-hydroxydecanoic acid moiety, compared to predicted ^13^C chemical shifts (green). (B) Experimental ^1^H (red) and ^13^C (blue) chemical shifts of the xantholysin variant 2 3-hydroxydodec-5-enoic acid moiety, compared to predicted ^13^C chemical shifts (green) assuming a *cis*-configuration. (C) Predicted ^13^C (green) chemical shifts of the xantholysin variant 2 3-hydroxydodec-5-enoic acid moiety assuming a *trans*-configuration. ^13^C chemical shift predictions were calculated using ChemNMR Pro 12.0 as implemented in the ChemDraw Ultra 12.0 software (*PerkinElmer, Inc.*). In the case of *cis*-3-hydroxydodec-5-enoic acid, the predicted ^13^C chemical shifts indicate a decrease in both the γ and ζ ^13^C chemical shifts compared to the HDA moiety (36.8 ppm→33.1 ppm and 29.3 ppm→27.7 ppm respectively). In the case of the *trans*-3-hydroxydodec-5-enoic acid, an increase in these same chemical shifts is predicted (36.8 ppm→39.1 ppm and 29.3 ppm→33.7 ppm respectively). In the *cis* configuration, the van der Waals radii of the hydrogens of the two carbons flanking the double bond are overlapping due to their spatial proximity, as opposed to the trans configuration. This steric compression causes an additional shielding and thus upfield shift of the associated carbons, which is not present in the case of the *trans* configuration (for reference, see Breitmaier E., Voelter E. (1987) Carbon-13 NMR Spectroscopy: High-Resolution Methods and Applications in Organic Chemistry and Biochemistry, 3^rd^ edition. Weinheim: VCH Verlagsgesellschaft mbH. 515 p.). The experimental values of the 3-hydroxydodec-5-enoic acid moiety compared to those of the HDA moiety (37.91 ppm→35.53 ppm and 29.60 ppm→27.28 ppm for the γ and ζ ^13^C nuclei respectively) show that the ^13^C chemical shifts correspond significantly better to the case of the *cis*-configuration, indeed showing a decrease. Therefore, this provides a significant indication for the lipid tail in xantholysin variant 2 to be in the *cis*-configuration.
(PDF)

Table S1 **Characterization of antibacterial activity of** ***P. putida*** **BW11M1.** Homology of genes disrupted in BW11M1 plasposon mutants lacking antibacterial activity using *X. alfalfae* subsp. *alfalfae* LMG 497 as indicator strain.
(PDF)

Table S2 ***In silico*** **analysis of adenylation domains in xantholysin synthetases.** Predicted substrate specificity of the A domains in the xantholysin NRPSs.
(PDF)

Table S3 **Antagonistic activity spectrum of** ***P. putida*** **BW11M1 wild type (WT) and xantholysin-deficient mutants.** The indicators shown are listed in Table 1 and Table S4. All other bacteria listed in Table S4 are not inhibited by strain BW11M1. Antagonism was semi-quantified by measuring the halo radii (in mm; average of three repeats; ND, no halo detected). Values in parentheses denote formation of a turbid halo. For some wild-type *Xanthomonas* strains a larger turbid halo was observed around a smaller clear halo. Additional italicized values in parentheses refer to the presence of a ‘secondary’ turbid growth inhibition halo.
(PDF)

Table S4 **Analysis of bacterial susceptibility to** ***P. putida*** **BW11M1.** List of non-*Xanthomonas* bacteria used as indicators.
(PDF)

Table S5 **Antifungal activity of** ***P. putida*** **BW11M1 and xantholysin-deficient mutants.** The effect on mycelial growth near a bacterial colony is indicated: (+) inhibition by wild type; (↓) or (−), reduced or no inhibition by *xtl* mutants compared to WT (as observed on PTA medium). For a particular indicator, the same phenotype was observed for the *xtlA*, *xtlB*, *xtlC* and *xtlR* mutants (CMPG2183, CMPG2187, CMPG2198, and CMPG2201, respectively), collectively designated *xtl* mutants. The inhibitory patterns for selected fungal strains are shown in Fig. S5.
(PDF)

Table S6 **NMR analysis of xantholysin A.** ^1^H and ^13^C NMR assignment of xantholysin A in DMF-d7 solution, 55°C.
(PDF)
