# Supplementary material for: The Antimicrobial Compound Xantholysin Defines a New Group of Pseudomonas Cyclic Lipopeptides
Source: PLoS One. 2013 May 17;8(5):e62946. doi: 10.1371/journal.pone.0062946 (PMC3656897; doi:10.1371/journal.pone.0062946)
Supplement: Figure S1 — Cladogram representation of a maximum-likelihood tree inferred from amino acid sequence alignment of condensation domains extracted from characterized Pseudomonas NRPSs. The lipopeptide-specific codes are specified in Fig. 3, with the xantholysin-related ones in bold. Clusters are differentiated according to the type of C-domain by color: conventional domains (green), dual epimerization/condensation domains (blue), and N-acylating starter domains (red). The tree was rooted with the divergent C1 domain of SyrE (in black). (PDF) [file pone.0062946.s001.pdf]

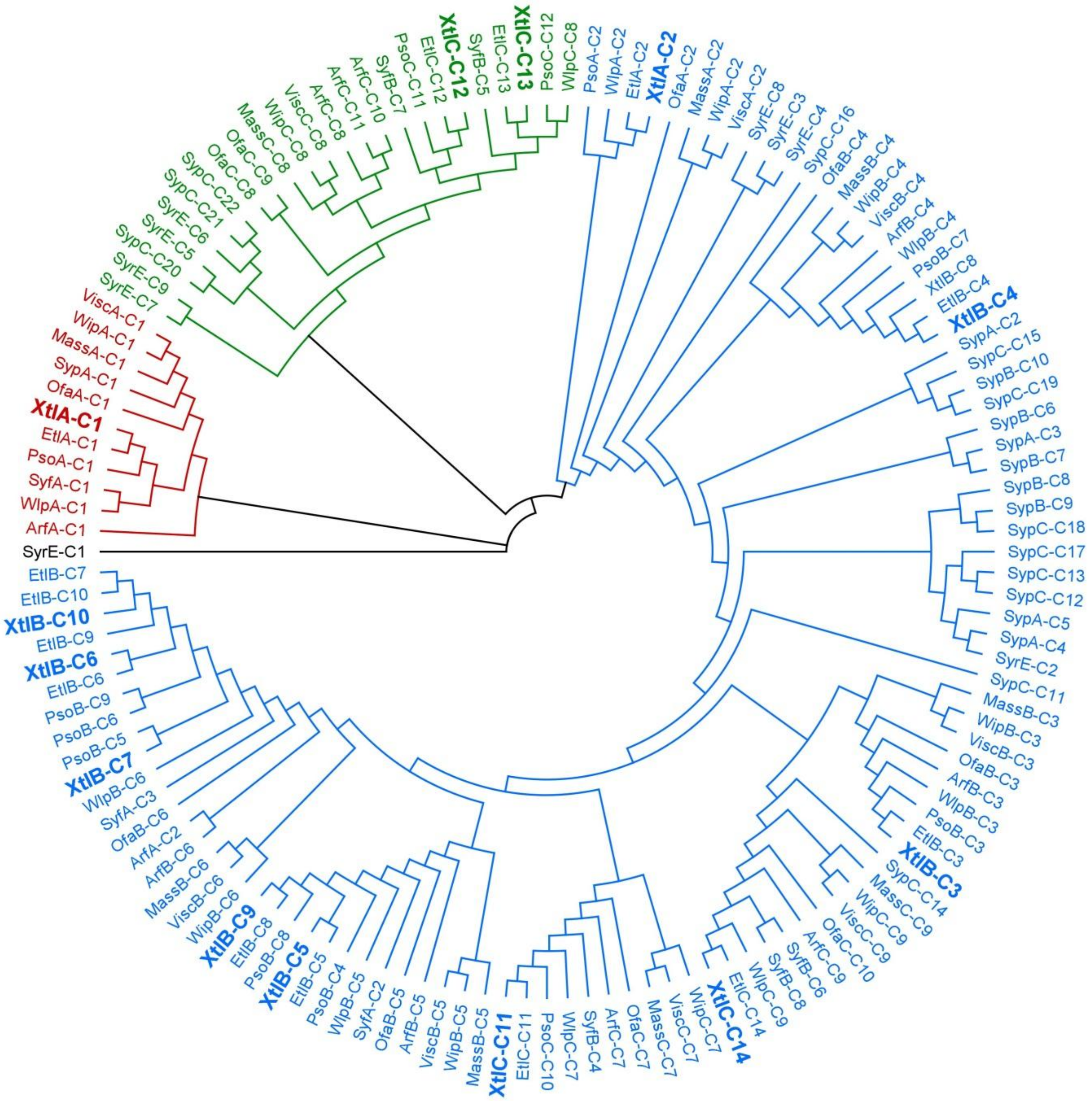

Figure S1. Cladogram representation of a maximum-likelihood tree inferred from amino acid sequence alignment of condensation domains extracted from characterized *Pseudomonas* NRPSs. The lipopeptide-specific codes are specified in Fig. 3, with the xantholysin-related ones in bold. Clusters are differentiated according to the type of C-domain by color: conventional domains (green), dual epimerization/condensation domains (blue), and N-acylating starter domains (red). The tree was rooted with the divergent C1 domain of SyrE (black).
