# Supplementary material for: The Antimicrobial Compound Xantholysin Defines a New Group of Pseudomonas Cyclic Lipopeptides
Source: PLoS One. 2013 May 17;8(5):e62946. doi: 10.1371/journal.pone.0062946 (PMC3656897; doi:10.1371/journal.pone.0062946)
Supplement: Figure S2 — Maximum-likelihood tree of thioesterase domains of characterized LP-producing Pseudomonas NRPSs. The clusters with tandemly organized domains TE1 (red) and TE2 (green) are shown in different colors. Lipopeptide-specific codes as in Fig. 3, with the xantholysin-related ones in bold. The tandem-TE domains of lysobactin synthetase LybB from Lysobacter sp. ATCC 53042 and the mono-TE domain of syringomycin synthetase SyrE from P. syringae pv. syringae B301D are included for comparison. The internal residue involved in cyclization with the carboxyterminal amino acid is specified for each TE1/TE domain (NA = not applicable for the linear syringafactins; OH-Phe = β-hydroxy phenylalanine). The scale bar represents 0.5 substitutions per site. (PDF) [file pone.0062946.s002.pdf]

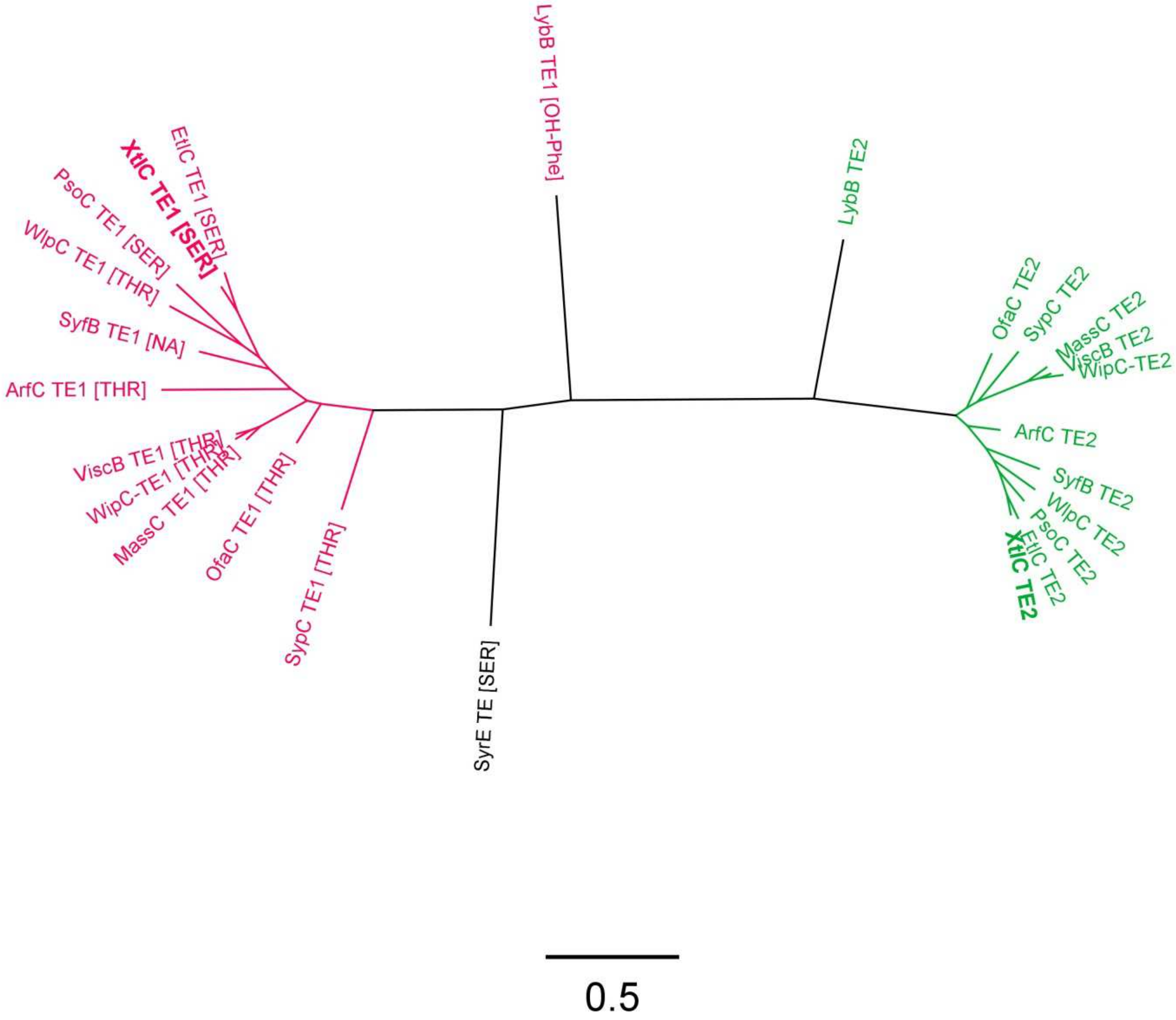

Figure S2. Maximum-likelihood tree of thioesterase domains of characterized LP-producing *Pseudomonas* NRPSs. The clusters with tandemly organized domains TE1 (red) and TE2 (green) are shown in different colors. Lipopeptide-specific codes as in Fig. 3, with the xantholysin-related ones in bold. The tandem-TE domains of lysobactin synthetase LybB from *Lysobacter* sp. ATCC 53042 and the mono-TE domain of syringomycin synthetase SyrE from *P. syringae* pv. *syringae* B301D are included for comparison. The internal residue involved in cyclization with the carboxyterminal amino acid is specified for each TE1/TE domain (NA = not applicable for the linear syringafactins; OH-Phe = β-hydroxy phenylalanine). The scale bar represents 0.5 substitutions per site.
