# Supplementary material for: The Antimicrobial Compound Xantholysin Defines a New Group of Pseudomonas Cyclic Lipopeptides
Source: PLoS One. 2013 May 17;8(5):e62946. doi: 10.1371/journal.pone.0062946 (PMC3656897; doi:10.1371/journal.pone.0062946)
Supplement: Figure S3 — Comparison of cognate regulators and associated export systems in lipopeptide-synthesizing Pseudomonas . Maximum-likelihood trees inferred from amino acid sequence alignments of (A) LuxR-family regulators encoded by genes linked with the respective initiatory NRPS genes and (B) the components of tripartite MacA/MacB/OprM-like export systems (concatenated sequences; same phylogeny was obtained for the three individual sequence sets (data not shown)). Lipopeptide-specific codes as in Fig. 3, with the xantholysin-related ones in bold. The regulatory gene encoding SyrF (panel A) is located downstream of syrE. In panel B, the Syf and Arf systems are not included (no sequences available for the corresponding OprM-like protein). The scale bars represent 0.09 (panel A) and 0.04 (panel B) substitutions per site. The xantholysin cluster (blue) and viscosin cluster (green) are shown in different colors. (PDF) [file pone.0062946.s003.pdf]

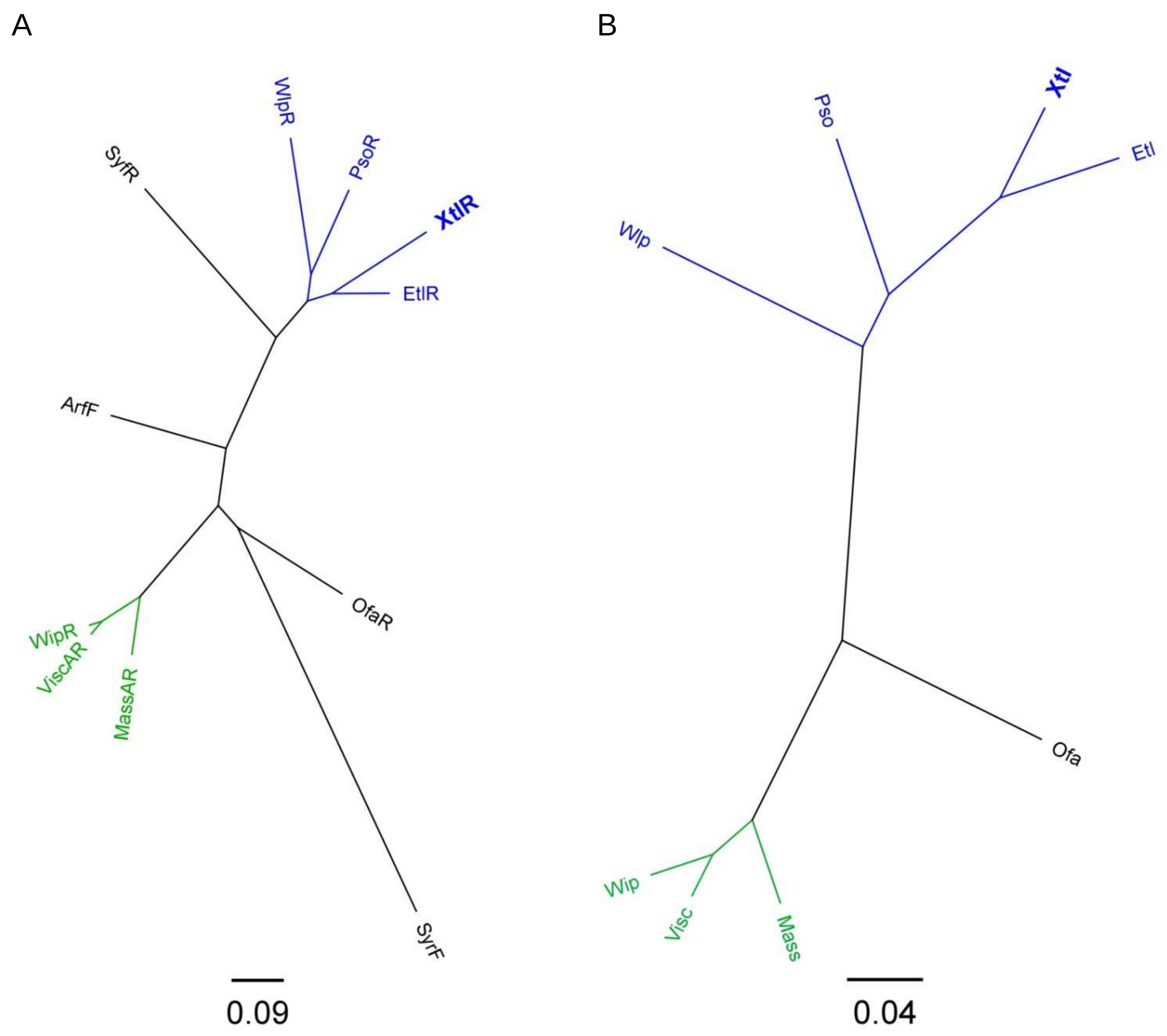


**Figure S3. Comparison of cognate regulators and associated export systems in lipopeptide-synthesizing *Pseudomonas*.** Maximum-likelihood trees inferred from amino acid sequence alignments of (A) LuxR-family regulators encoded by genes linked with the respective initiatory NRPS genes and (B) the components of tripartite MacA/MacB/OprM-like export systems (concatenated sequences; same phylogeny was obtained for the three individual sequence sets (data not shown)). Lipopeptide-specific codes as in Fig. 3, with the xantholysin-related ones in bold. The regulatory gene encoding SyrF (panel A) is located downstream of *syrE*. In panel B, the Syf and Arf systems are not included (no sequences available for the corresponding OprM-like protein). The scale bars represent 0.09 (panel A) and 0.04 (panel B) substitutions per site. The xantholysin cluster (blue) and viscosin cluster (green) are shown in different colors.
