# Supplementary material for: The Antimicrobial Compound Xantholysin Defines a New Group of Pseudomonas Cyclic Lipopeptides
Source: PLoS One. 2013 May 17;8(5):e62946. doi: 10.1371/journal.pone.0062946 (PMC3656897; doi:10.1371/journal.pone.0062946)
Supplement: Figure S4 — Growth inhibition of representative indicator bacteria by purified xantholysin. 10 µl-samples of purified xantholysin in methanol, containing 18 µg (A) or 9 µg (B) were spotted on an agar plate and overlaid with indicator cells after solvent evaporation. Methanol control spots did not cause growth inhibition. (PDF) [file pone.0062946.s004.pdf]

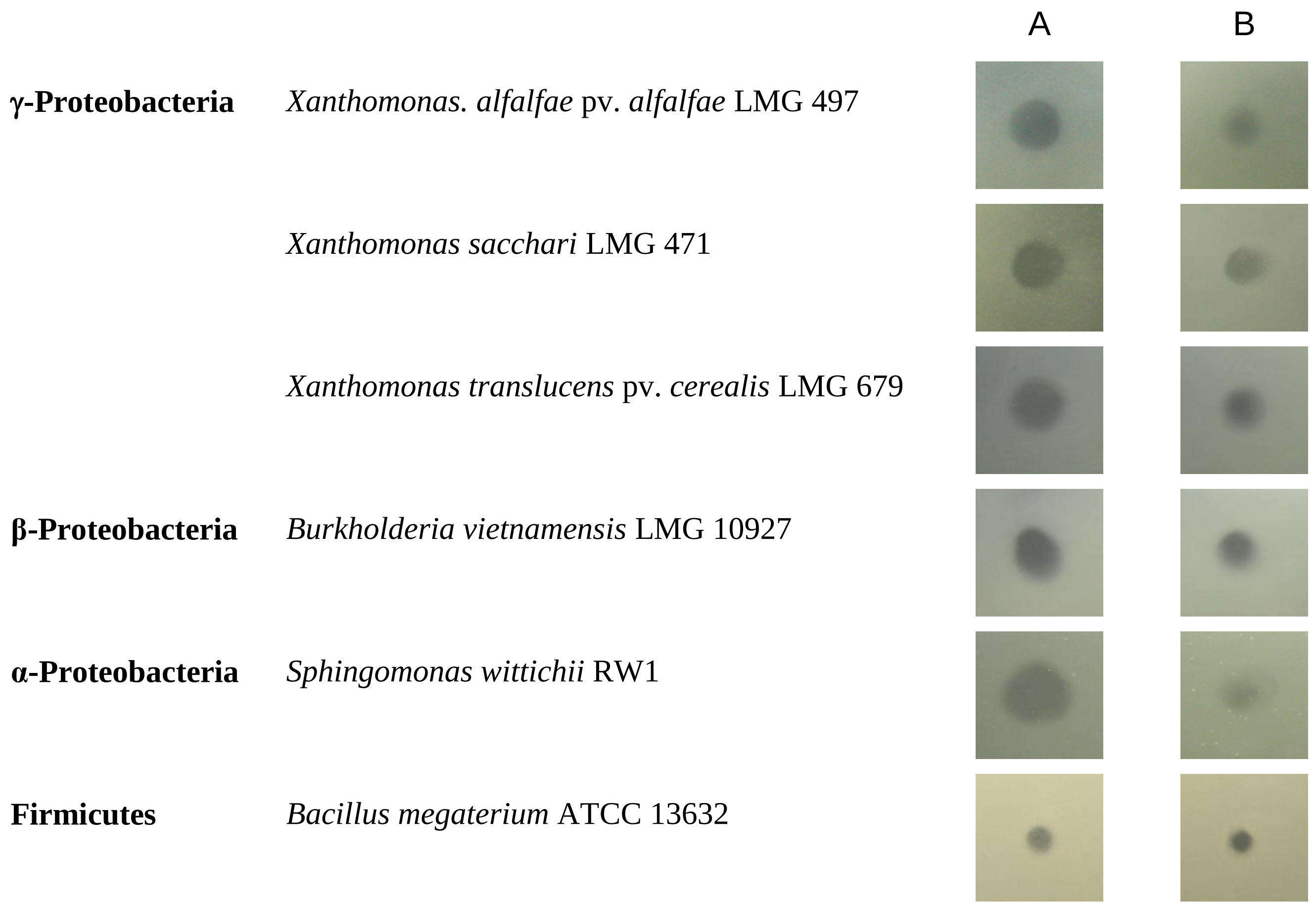

Figure S4. Growth inhibition of representative indicator bacteria by purified xantholysin. 10 μl-samples of purified xantholysin in methanol, containing 18 μg (A) or 9 μg (B) were spotted on an agar plate and overlaid with indicator cells after solvent evaporation. Methanol control spots did not cause growth inhibition.
