# Supplementary material for: The Antimicrobial Compound Xantholysin Defines a New Group of Pseudomonas Cyclic Lipopeptides
Source: PLoS One. 2013 May 17;8(5):e62946. doi: 10.1371/journal.pone.0062946 (PMC3656897; doi:10.1371/journal.pone.0062946)
Supplement: Figure S5 — Antifungal activity of xantholysin. Growth inhibition of fungi by P. putida BW11M1 (WT) and xantholysin-deficient mutants (xtlA, xtlB, xtlC, xtlR; CMPG2183, CMPG2187, CMPG2198, and CMPG2201, respectively). The application pattern of bacterial cell suspensions spotted around a centrally positioned plug with fungal mycelium is shown schematically in (A). (B) Alternaria longipes CBS 620.83, (C) Ascochyta pisi MUCL30164, (D) Botrytis cinerea MUCL30158, (E) Colletotrichum gloeosporoides SR24, (F) Fusarium culmorum MUCL30162, (G) Fusarium graminearum MUCL30161, (H) Fusarium oxysporum MUCL909, (I) Fusarium oxysporum MUCL30160, (J) Fusarium oxysporum f. sp. radices-lycopersi CBS 873.95, (K) Fusarium oxysporum f. sp. vasinfectum CBS 410.90, (L) Gloeosporium musarum MUCL500, (M) Gloesporium solani CBS 194.32, (N) Nectria haematococca MUCL20259, (O) Pyrenophora tritici-repentis MUCL30217, (P) Rhizoctonia solani CBS 211.84. (PDF) [file pone.0062946.s005.pdf]

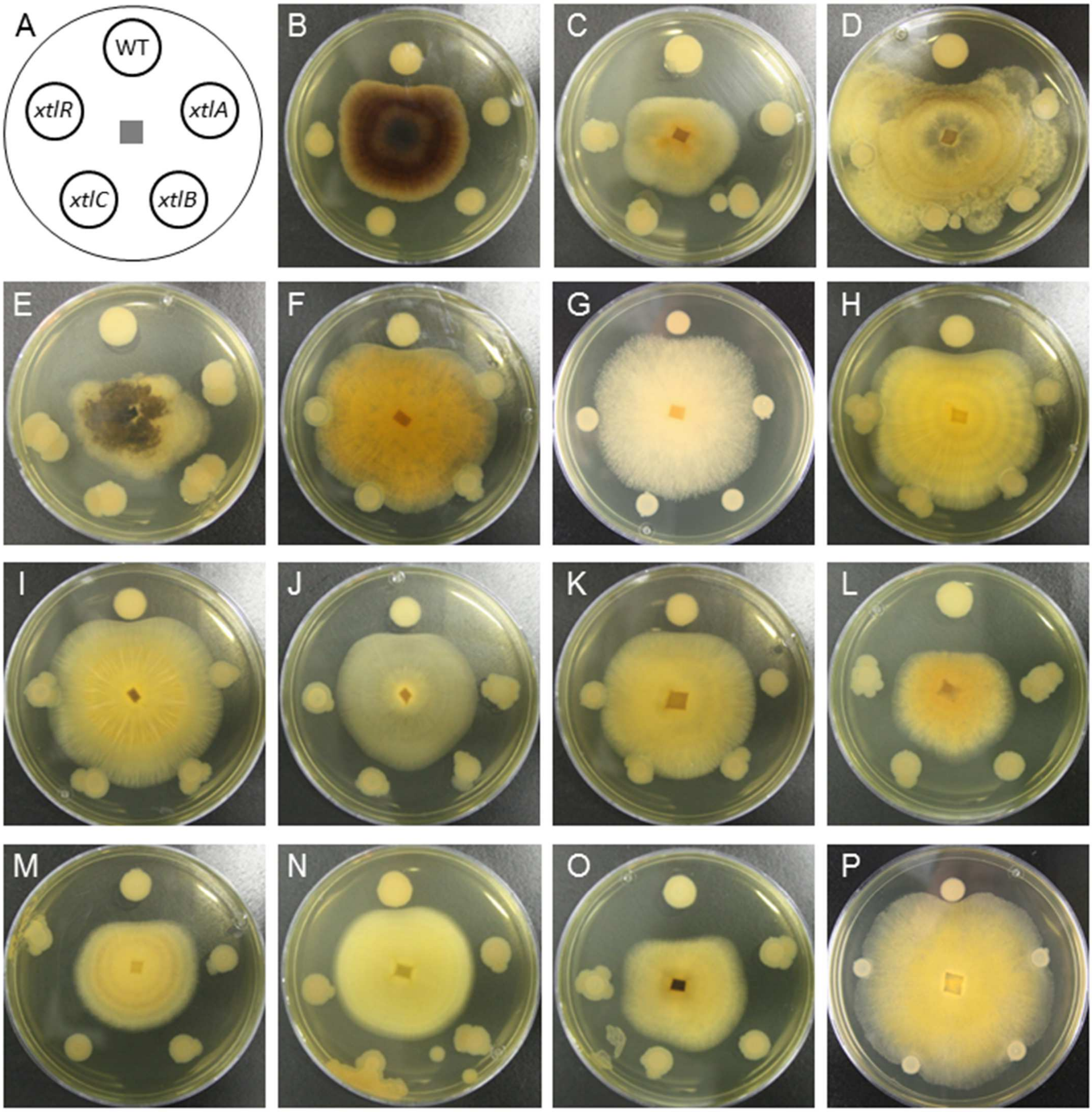

Figure S5. Antifungal activity of xantholysin. Growth inhibition of fungi by *P. putida* BW11M1 (WT) and xantholysin-deficient mutants (*xtlA*, *xtlB*, *xtlC*, *xtlR;* CMPG2183, CMPG2187, CMPG2198, and CMPG2201, respectively). The application pattern of bacterial cell suspensions spotted around a centrally positioned plug with fungal mycelium is shown schematically in (A). (B) *Alternaria longipes* CBS 620.83, (C) *Ascochyta pisi* MUCL30164, (D) *Botrytis cinerea* MUCL30158, (E) *Colletotrichum gloeosporoides* SR24, (F) *Fusarium culmorum* MUCL30162, (G) *Fusarium graminearum* MUCL30161, (H) *Fusarium oxysporum* MUCL909, (I) *Fusarium oxysporum* MUCL30160, (J) *Fusarium oxysporum* f. sp. *radicis – lycopersi* CBS 873.95, (K) *Fusarium oxysporum* f. sp. *vasinfectum* CBS 410.90, (L) *Gloeosporium musarum* MUCL500, (M) *Gloesporium solani* CBS 194.32, (N) *Nectria haematococca* MUCL20259, (O) *Pyrenophora tritici-repentis* MUCL30217, (P) *Rhizoctonia solani* CBS 211.84.
