# Supplementary material for: The Antimicrobial Compound Xantholysin Defines a New Group of Pseudomonas Cyclic Lipopeptides
Source: PLoS One. 2013 May 17;8(5):e62946. doi: 10.1371/journal.pone.0062946 (PMC3656897; doi:10.1371/journal.pone.0062946)
Supplement: Figure S6 — Chromatographic separation of xantholysin congeners. LC-MS chromatogram of the extracted mixture prior to purification using UV-detection at 214 nm. (PDF) [file pone.0062946.s006.pdf]

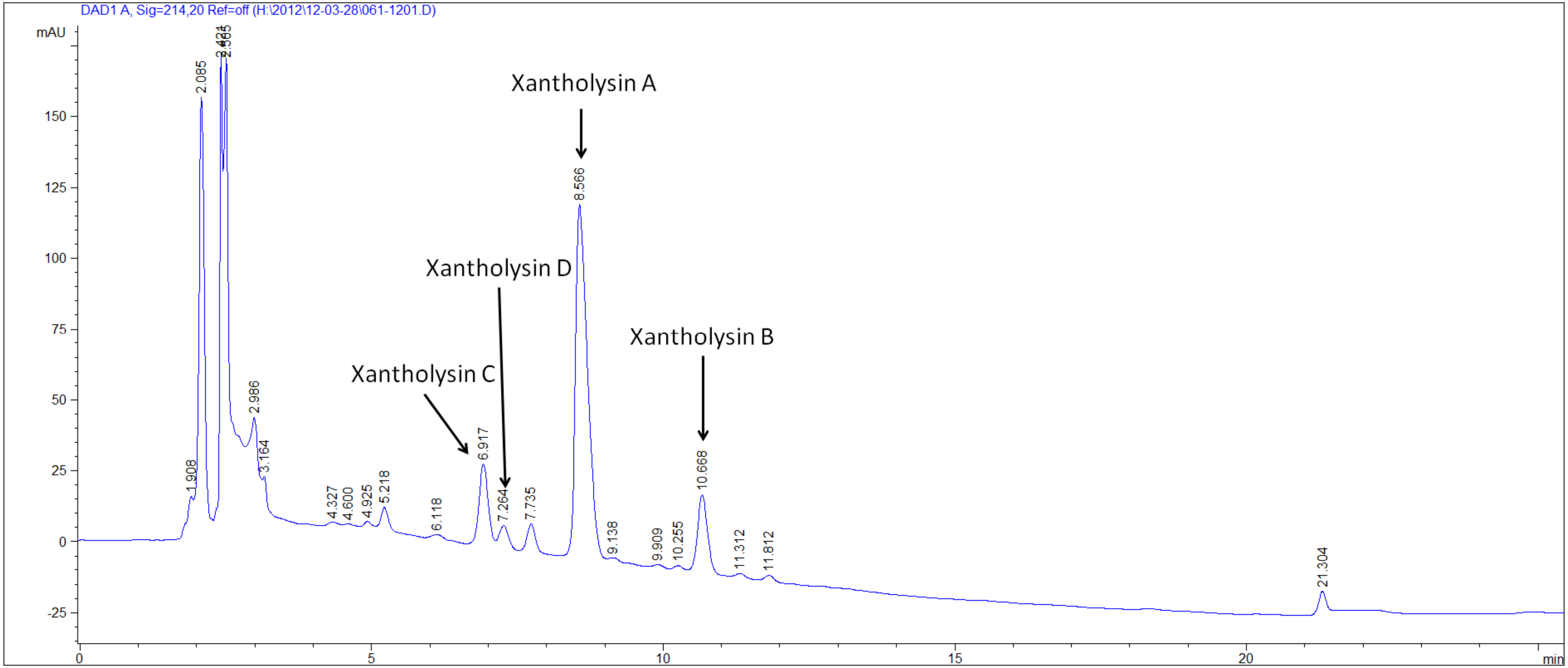

Figure S6. Chromatographic separation of xantholysin congeners. LC-MS chromatogram of the extracted mixture prior to purification using UV-detection at 214 nm.
