# Supplementary material for: The Antimicrobial Compound Xantholysin Defines a New Group of Pseudomonas Cyclic Lipopeptides
Source: PLoS One. 2013 May 17;8(5):e62946. doi: 10.1371/journal.pone.0062946 (PMC3656897; doi:10.1371/journal.pone.0062946)
Supplement: Figure S7 — Mass spectrometry of xantholysin congeners. Mass spectra of xantholysin A and its variants obtained during LC-MS analysis of the extracted mixture prior to purification. Preliminary experiments with a high-resolution instrument showed that the peaks represent ions with charge z = 2. (PDF) [file pone.0062946.s007.pdf]

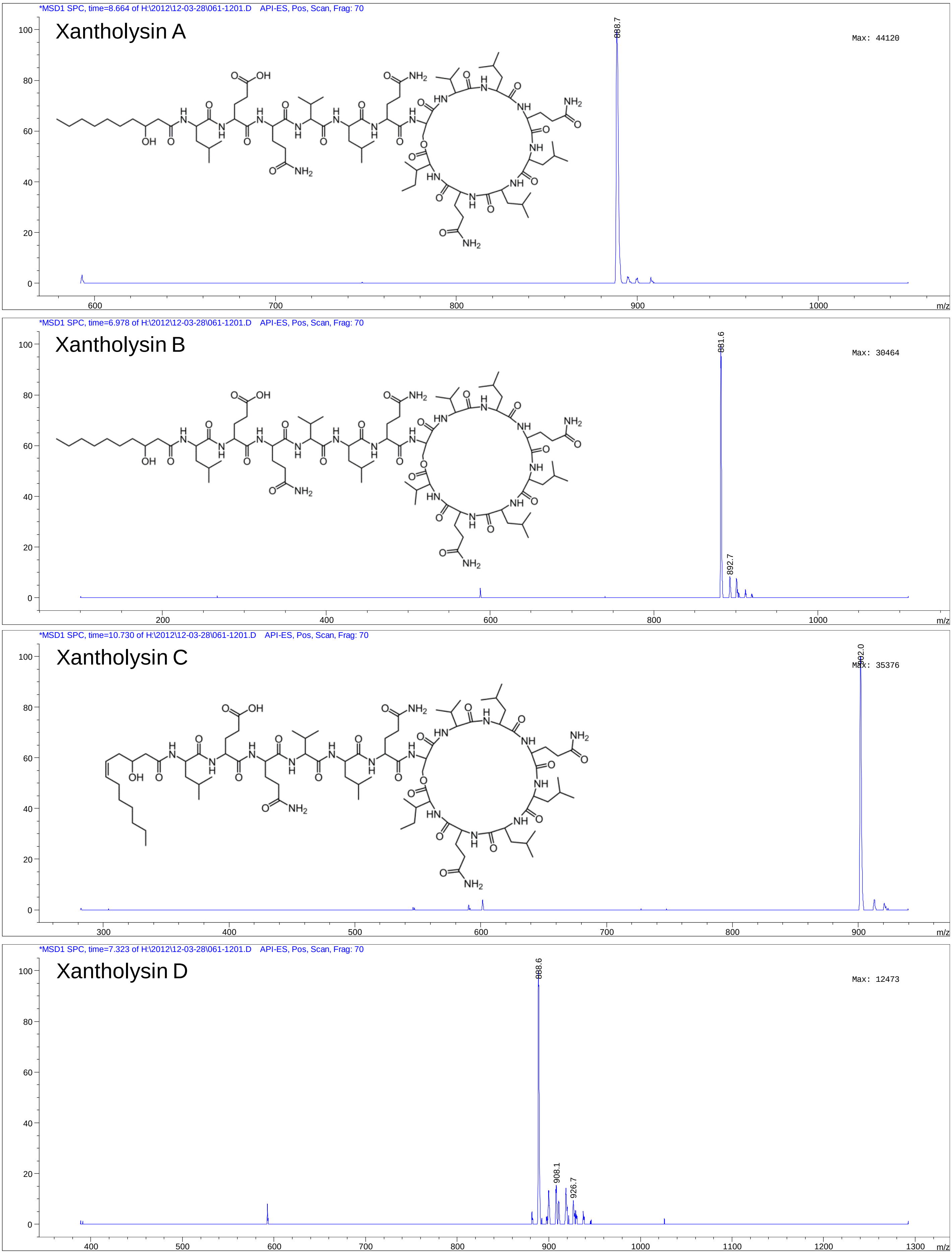

Figure S7. Mass spectrometry of xantholysin congeners. Mass spectra of xantholysin A and its variants obtained during LC-MS analysis of the extracted mixture prior to purification**.** Preliminary experiments with a high-resolution instrument showed that the peaks represent ions with charge z=2.
