# Supplementary material for: The Antimicrobial Compound Xantholysin Defines a New Group of Pseudomonas Cyclic Lipopeptides
Source: PLoS One. 2013 May 17;8(5):e62946. doi: 10.1371/journal.pone.0062946 (PMC3656897; doi:10.1371/journal.pone.0062946)
Supplement: Figure S9 — 2D NMR analysis of xantholysin A. 2D 1H-1H TOCSY spectrum of xantholysin A in DMF-d7 solution, 55°C, 500 MHz, revealing the characteristic amino acid spin system patterns. (PDF) [file pone.0062946.s009.pdf]

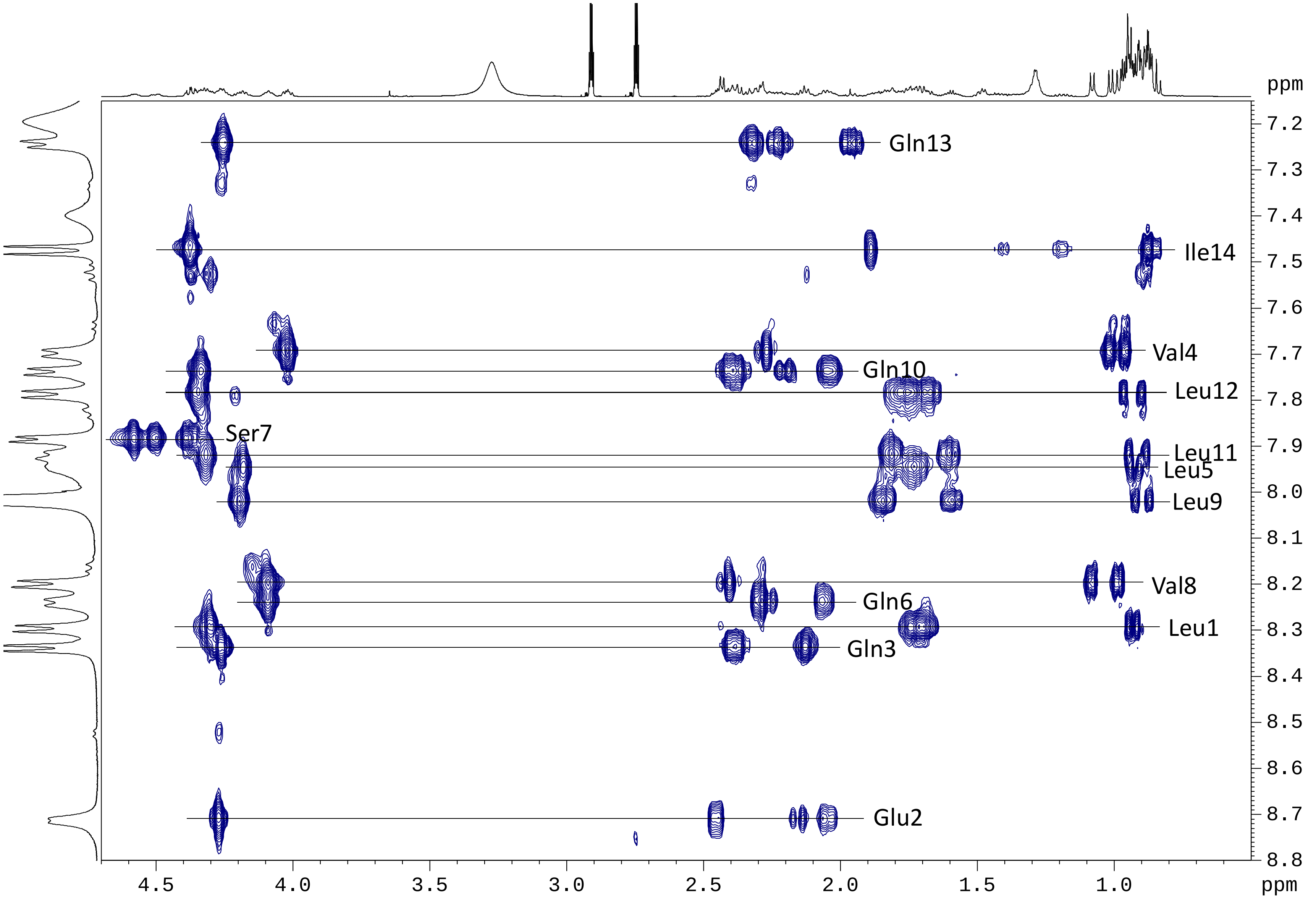

Figure S9. 2D NMR analysis of xantholysin A. 2D ^{1}H-^{1}H TOCSY spectrum of xantholysin A in DMF-d7 solution, 55°C, 500 MHz, revealing the characteristic amino acid spin system patterns.
