# Supplementary material for: The Antimicrobial Compound Xantholysin Defines a New Group of Pseudomonas Cyclic Lipopeptides
Source: PLoS One. 2013 May 17;8(5):e62946. doi: 10.1371/journal.pone.0062946 (PMC3656897; doi:10.1371/journal.pone.0062946)
Supplement: Figure S10 — Nuclear Overhauser effect spectroscopy of xantholysin A. Established NOE contacts in xantholysin A, observed in a 2D 1H-1H NOESY spectrum with 300 ms mixing time. (PDF) [file pone.0062946.s010.pdf]

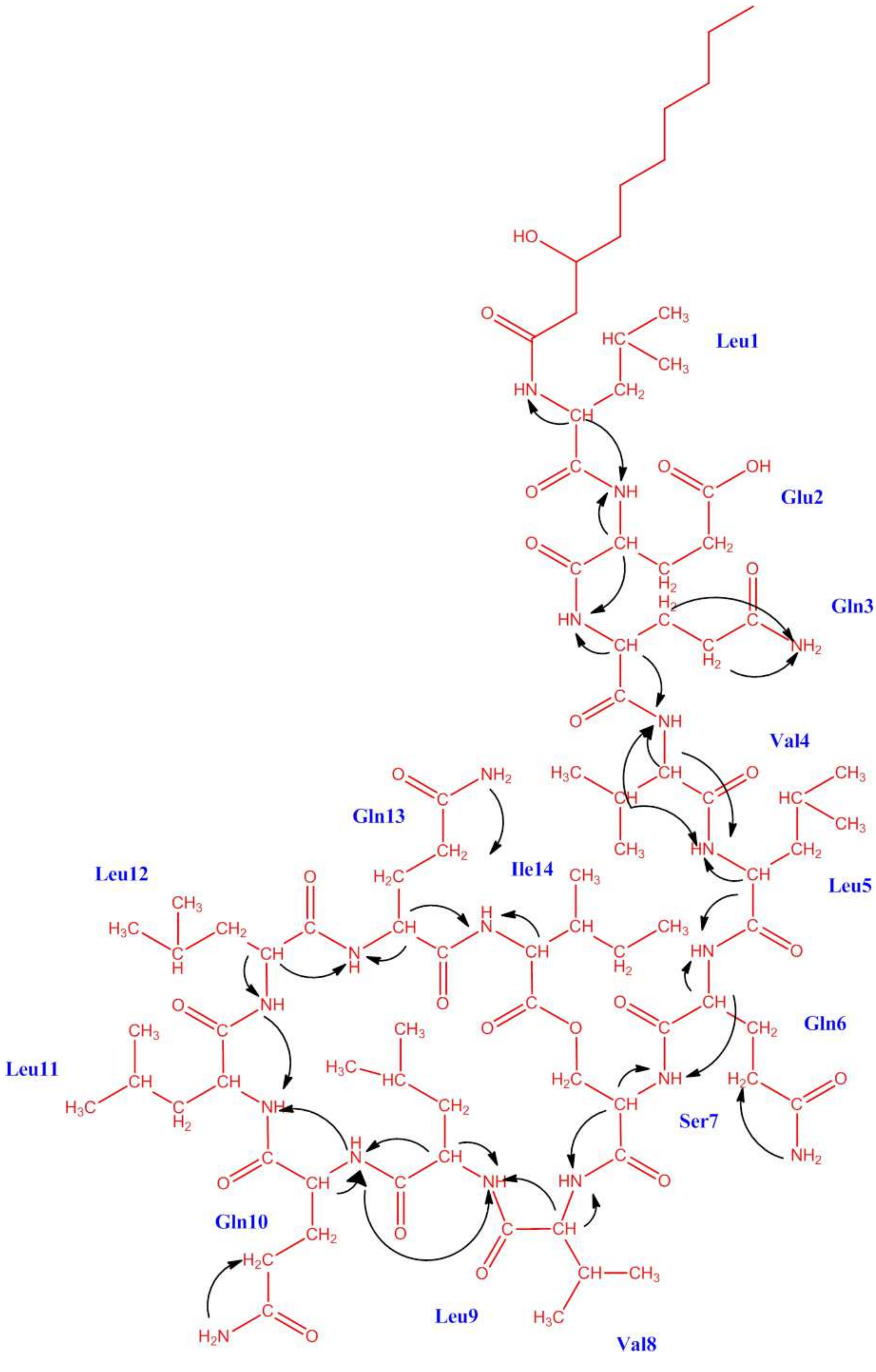

Figure S10. Nuclear Overhauser effect spectroscopy of xantholysin A. Established NOE contacts in xantholysin A, observed in a 2D ^{1}H-^{1}H NOESY spectrum with 300 ms mixing time.
