# Supplementary material for: The Antimicrobial Compound Xantholysin Defines a New Group of Pseudomonas Cyclic Lipopeptides
Source: PLoS One. 2013 May 17;8(5):e62946. doi: 10.1371/journal.pone.0062946 (PMC3656897; doi:10.1371/journal.pone.0062946)
Supplement: Figure S11 — High resolution mass spectrum of xantholysin A. (A) Full mass spectrum. (B) Zoom on the [M+H]+ molecular ion peaks. Expected exact mass of xantholysin A (C84H146N18O23)+H+: 1776.0881 Da; observed exact mass of xantholysin A+H+: 1776.0837 Da. (PDF) [file pone.0062946.s011.pdf]

**A**

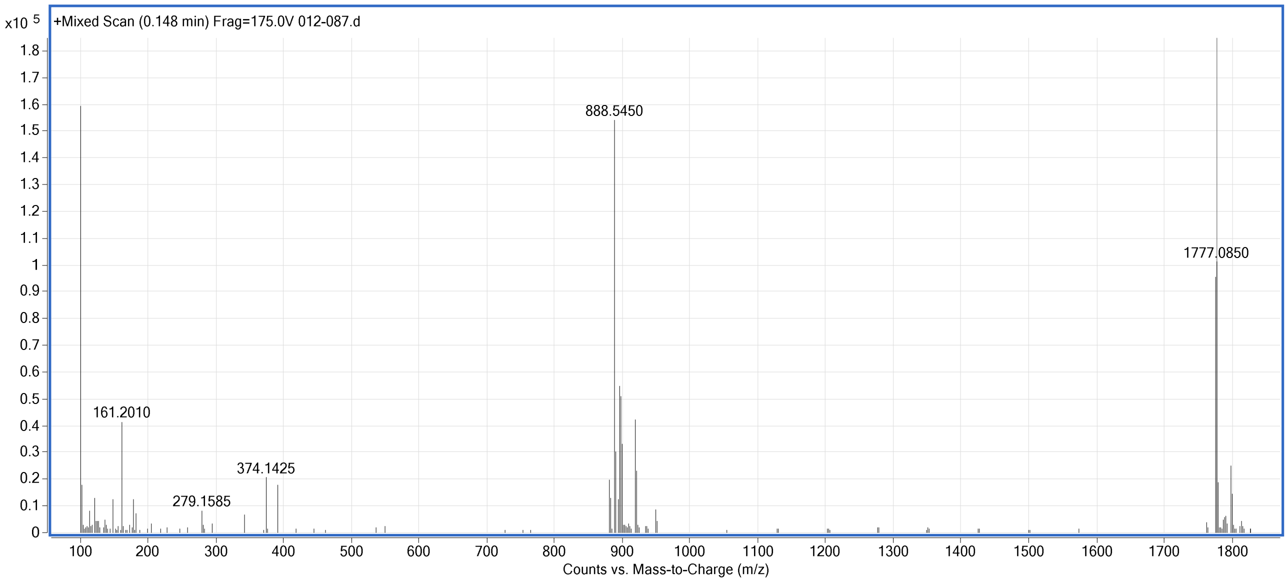


**B**

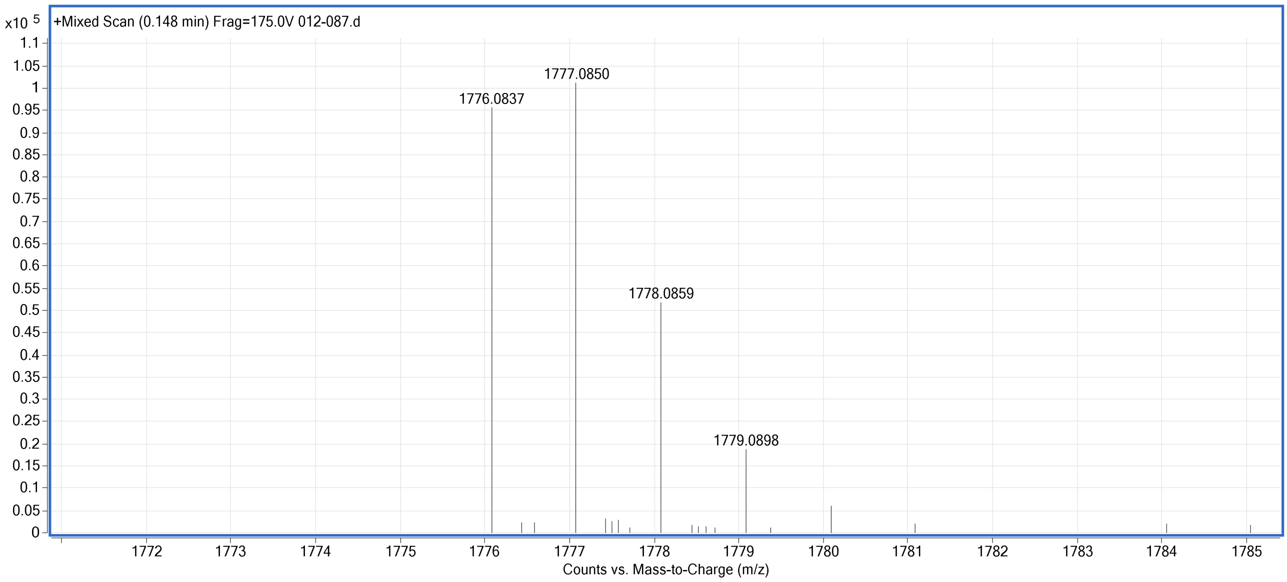


**Figure S11. High resolution mass spectrum of xantholysin A.** (A) Full mass spectrum. (B) Zoom on the $[M+H]^+$ molecular ion peaks. Expected exact mass of xantholysin A ($C_{84}H_{146}N_{18}O_{23}$) + $H^+$: 1776.0881 Da; observed exact mass of xantholysin A + $H^+$: 1776.0837 Da.

$$\Delta = \frac{1776.0881 - 1776.0837}{1776.0881} \times 10^6 \text{ppm} = 2.5\text{ppm}$$
