# Supplementary material for: The Antimicrobial Compound Xantholysin Defines a New Group of Pseudomonas Cyclic Lipopeptides
Source: PLoS One. 2013 May 17;8(5):e62946. doi: 10.1371/journal.pone.0062946 (PMC3656897; doi:10.1371/journal.pone.0062946)
Supplement: Figure S12 — 1D NMR analysis of xantholysin B. 1D 1H spectrum of xantholysin variant 1, in DMF-d7 solution, 55°C, 700 MHz. (PDF) [file pone.0062946.s012.pdf]

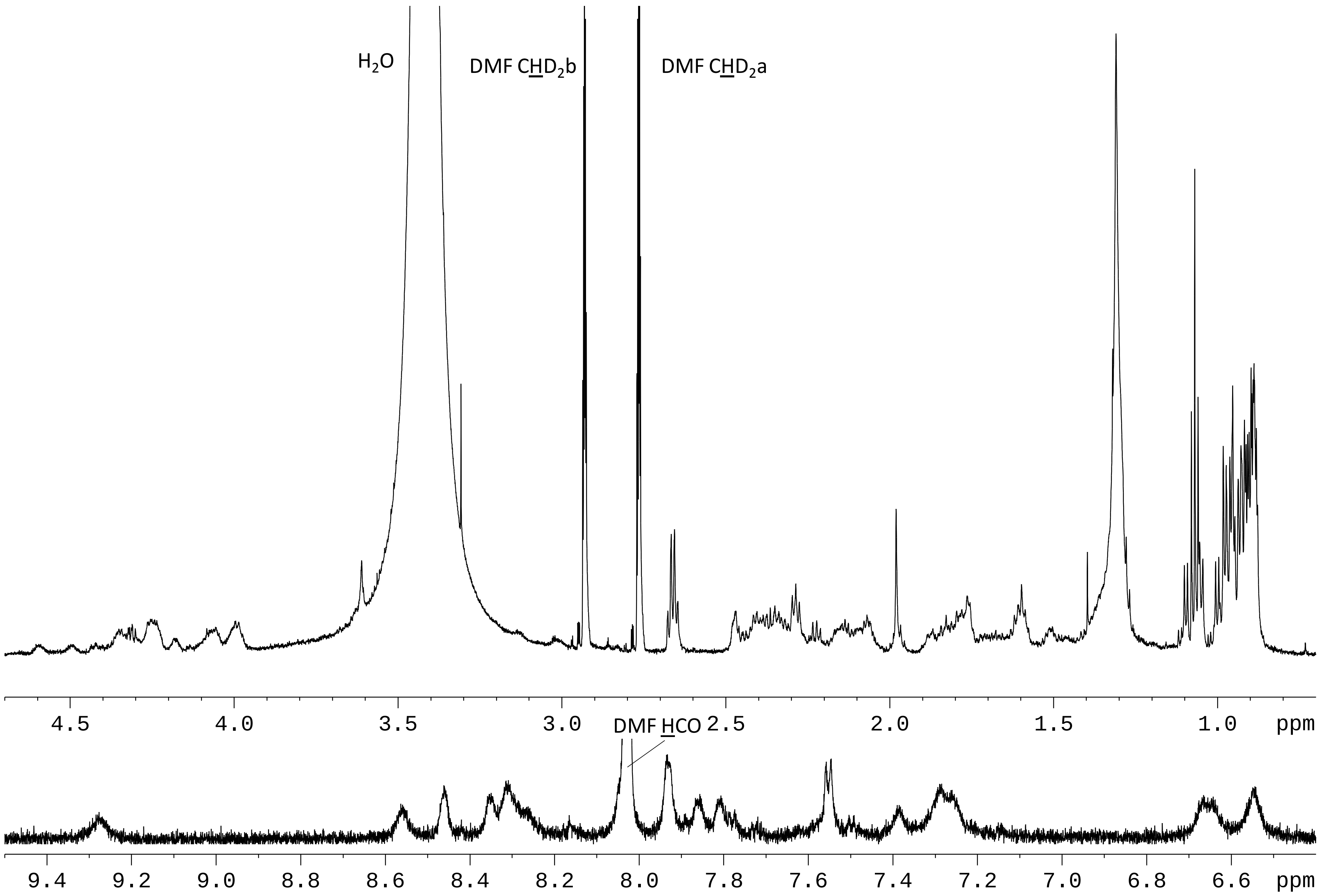

Figure S12. 1D NMR analysis of xantholysin B. 1D ^{1}H spectrum of xantholysin variant 1, in DMF-d7 solution, 55°C, 700 MHz.
