# Supplementary material for: The Antimicrobial Compound Xantholysin Defines a New Group of Pseudomonas Cyclic Lipopeptides
Source: PLoS One. 2013 May 17;8(5):e62946. doi: 10.1371/journal.pone.0062946 (PMC3656897; doi:10.1371/journal.pone.0062946)
Supplement: Figure S13 — 1D NMR analysis of xantholysin C. 1D 1H spectrum of xantholysin variant 2, in DMF-d7 solution, 55°C, 700 MHz. The inset shows the signals arising from the alkene protons of the 3-hydroxydodec-5-enoic acid moiety, on top of an unidentified resonance from an impurity. (PDF) [file pone.0062946.s013.pdf]

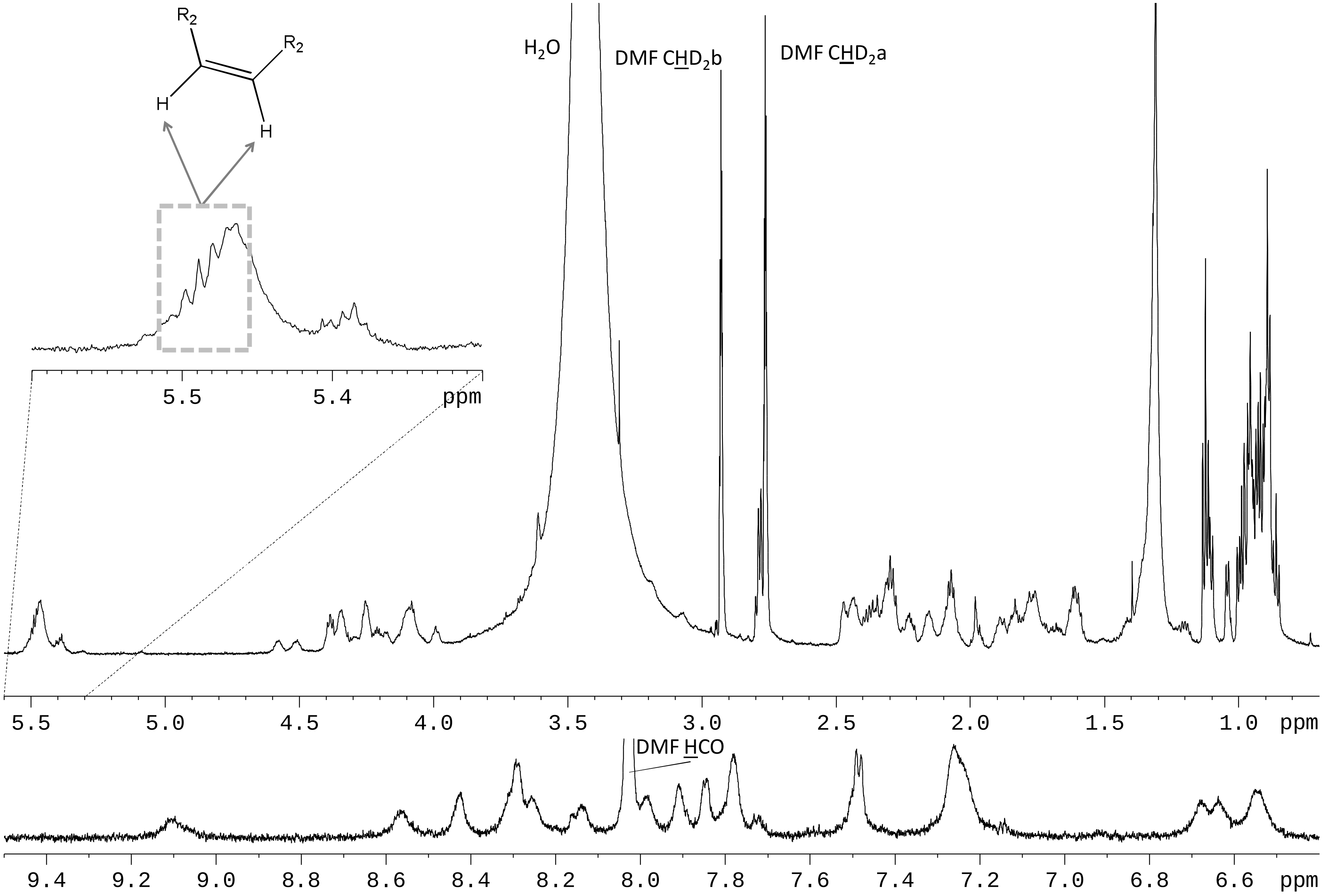

Figure S13. 1D NMR analysis of xantholysin C. 1D ^{1}H spectrum of xantholysin variant 2, in DMF-d7 solution, 55°C, 700 MHz. The inset shows the signals arising from the alkene protons of the 3-hydroxydodec-5-enoic acid moiety, on top of an unidentified resonance from an impurity.
