# Supplementary material for: The Antimicrobial Compound Xantholysin Defines a New Group of Pseudomonas Cyclic Lipopeptides
Source: PLoS One. 2013 May 17;8(5):e62946. doi: 10.1371/journal.pone.0062946 (PMC3656897; doi:10.1371/journal.pone.0062946)
Supplement: Figure S15 — High resolution mass spectrum of xantholysin B. (A) Full mass spectrum. (B) Zoom on the [M+H]+ and [M+Na]+ molecular ion peaks. Expected exact mass of xantholysin B (C83H144N18O23)+H+: 1762.0724 Da; observed exact mass of xantholysin B+H+: 1762.0647 Da. (PDF) [file pone.0062946.s015.pdf]

A

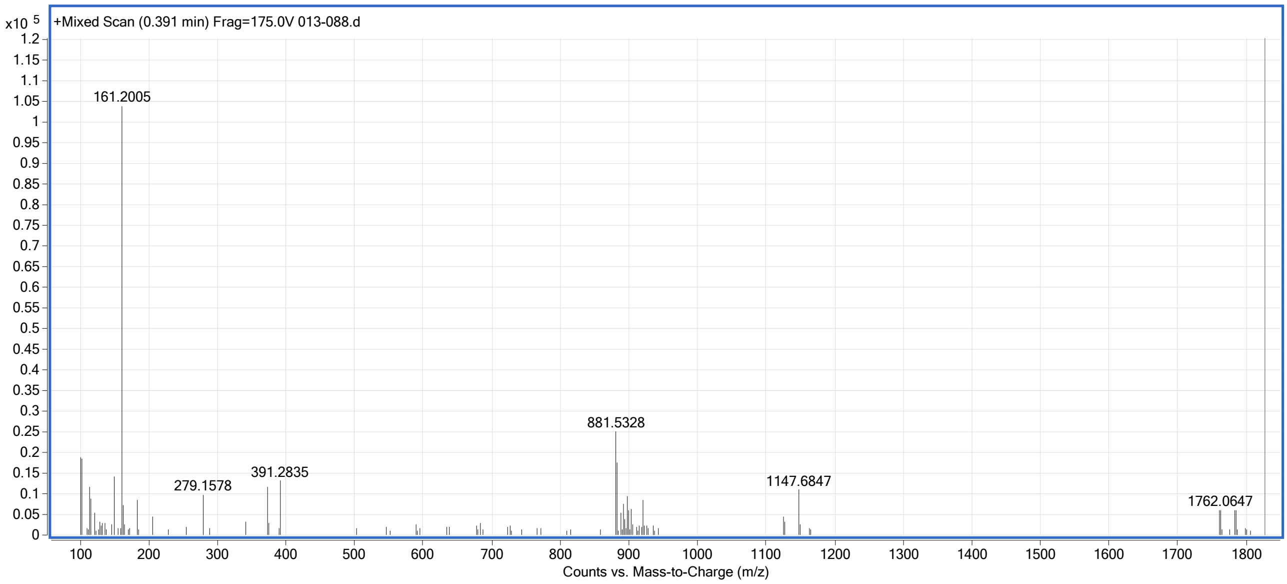

B

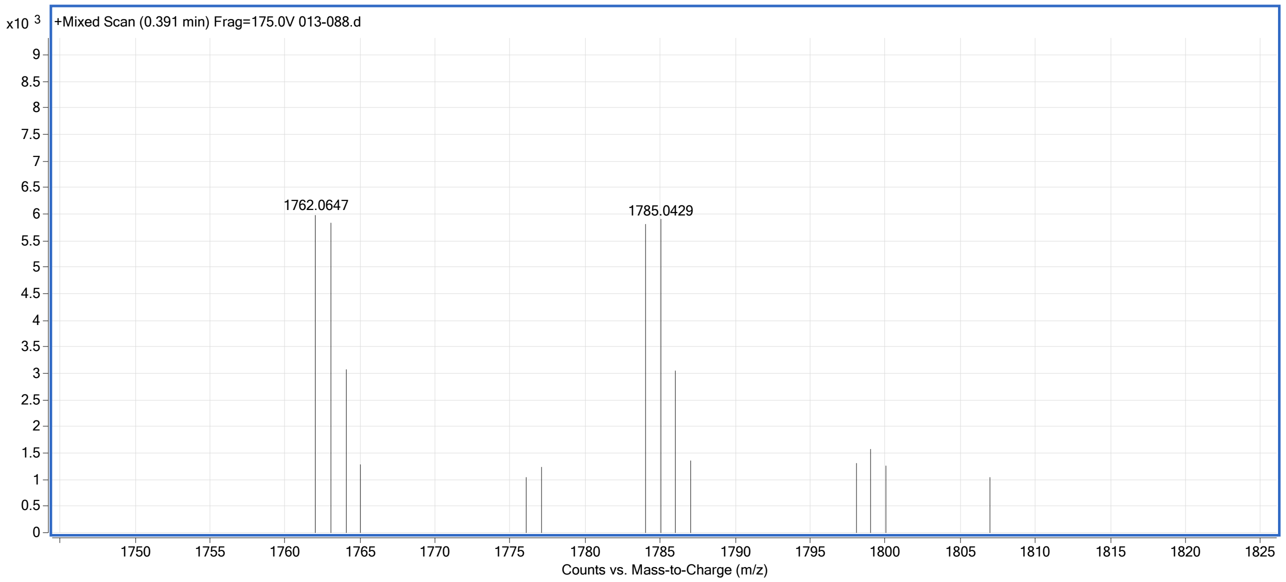

Figure S15. High resolution mass spectrum of xantholysin B. (A) Full mass spectrum. (B) Zoom on the $[M+H]^+$ and $[M+Na]^+$ molecular ion peaks. Expected exact mass of xantholysin B ($C_{83}H_{144}N_{18}O_{23}$) + H^+: 1762.0724 Da; observed exact mass of xantholysin B + H^+: 1762.0647 Da.

$$\Delta = \frac{1762.0724 - 1762.0647}{1762.0724} \times 10^6 \text{ppm} = 4.4\text{ppm}$$
