# Supplementary material for: The Antimicrobial Compound Xantholysin Defines a New Group of Pseudomonas Cyclic Lipopeptides
Source: PLoS One. 2013 May 17;8(5):e62946. doi: 10.1371/journal.pone.0062946 (PMC3656897; doi:10.1371/journal.pone.0062946)
Supplement: Figure S16 — High resolution mass spectrum of xantholysin C. (A) Full mass spectrum. (B) Zoom on the [M+H]+ and [M+Na]+ molecular ion peaks. Expected exact mass of xantholysin C (C86H148N18O23)+H+: 1802.1037 Da; observed exact mass of xantholysin C+H+: 1802.0973 Da. (PDF) [file pone.0062946.s016.pdf]

A

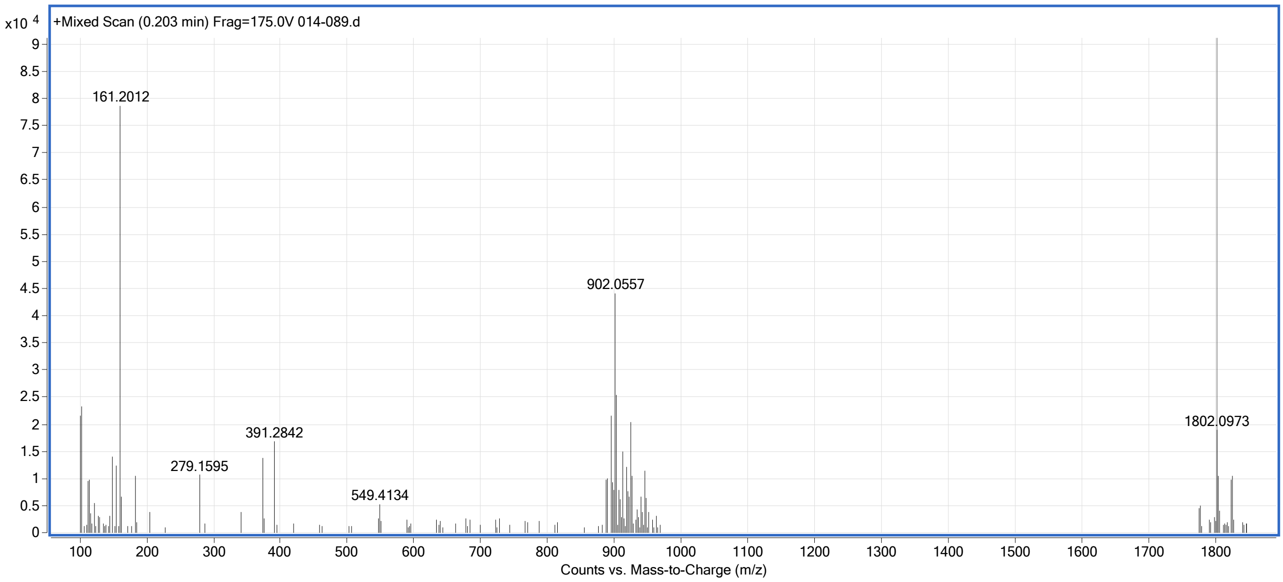

B

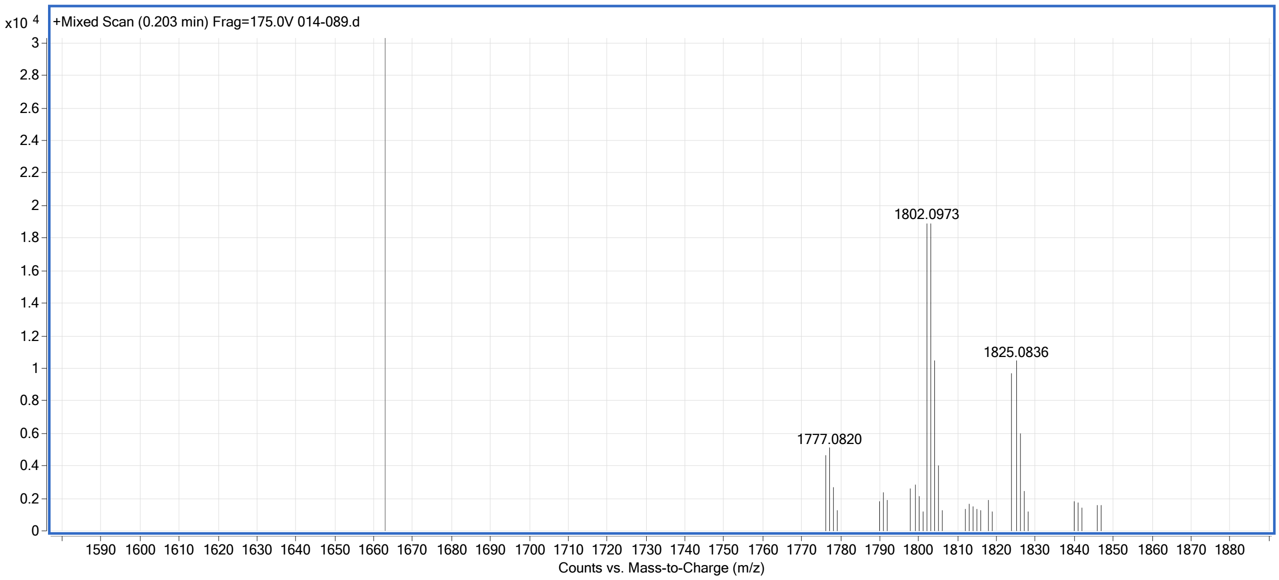

Figure S16. High resolution mass spectrum of xantholysin C. (A) Full mass spectrum. (B) Zoom on the $[M+H]^+$ and $[M+Na]^+$ molecular ion peaks. Expected exact mass of xantholysin C ($C_{86}H_{148}N_{18}O_{23}$) + H^+: 1802.1037 Da; observed exact mass of xantholysin C + H^+: 1802.0973 Da.

$$\Delta = \frac{1802.1037 - 1802.0973}{1802.1037} \times 10^6 \text{ppm} = 3.6\text{ppm}$$
