# Supplementary material for: The Antimicrobial Compound Xantholysin Defines a New Group of Pseudomonas Cyclic Lipopeptides
Source: PLoS One. 2013 May 17;8(5):e62946. doi: 10.1371/journal.pone.0062946 (PMC3656897; doi:10.1371/journal.pone.0062946)
Supplement: Figure S17 — NMR analysis of fatty acid moiety in xantholysin C. 1H and 13C chemical shift comparison between the lipid tail of xantholysin A and variant 2. (A) Experimental 1H (red) and 13C (blue) chemical shifts of the xantholysin 3-hydroxydecanoic acid moiety, compared to predicted 13C chemical shifts (green). (B) Experimental 1H (red) and 13C (blue) chemical shifts of the xantholysin variant 2 3-hydroxydodec-5-enoic acid moiety, compared to predicted 13C chemical shifts (green) assuming a cis-configuration. (C) Predicted 13C (green) chemical shifts of the xantholysin variant 2 3-hydroxydodec-5-enoic acid moiety assuming a trans-configuration. 13C chemical shift predictions were calculated using ChemNMR Pro 12.0 as implemented in the ChemDraw Ultra 12.0 software (PerkinElmer, Inc.). In the case of cis-3-hydroxydodec-5-enoic acid, the predicted 13C chemical shifts indicate a decrease in both the γ and ζ 13C chemical shifts compared to the HDA moiety (36.8 ppm→33.1 ppm and 29.3 ppm→27.7 ppm respectively). In the case of the trans-3-hydroxydodec-5-enoic acid, an increase in these same chemical shifts is predicted (36.8 ppm→39.1 ppm and 29.3 ppm→33.7 ppm respectively). In the cis configuration, the van der Waals radii of the hydrogens of the two carbons flanking the double bond are overlapping due to their spatial proximity, as opposed to the trans configuration. This steric compression causes an additional shielding and thus upfield shift of the associated carbons, which is not present in the case of the trans configuration (for reference, see Breitmaier E., Voelter E. (1987) Carbon-13 NMR Spectroscopy: High-Resolution Methods and Applications in Organic Chemistry and Biochemistry, 3rd edition. Weinheim: VCH Verlagsgesellschaft mbH. 515 p.). The experimental values of the 3-hydroxydodec-5-enoic acid moiety compared to those of the HDA moiety (37.91 ppm→35.53 ppm and 29.60 ppm→27.28 ppm for the γ and ζ 13C nuclei respectively) show that the 13C chemical shifts correspond signific [file pone.0062946.s017.pdf]

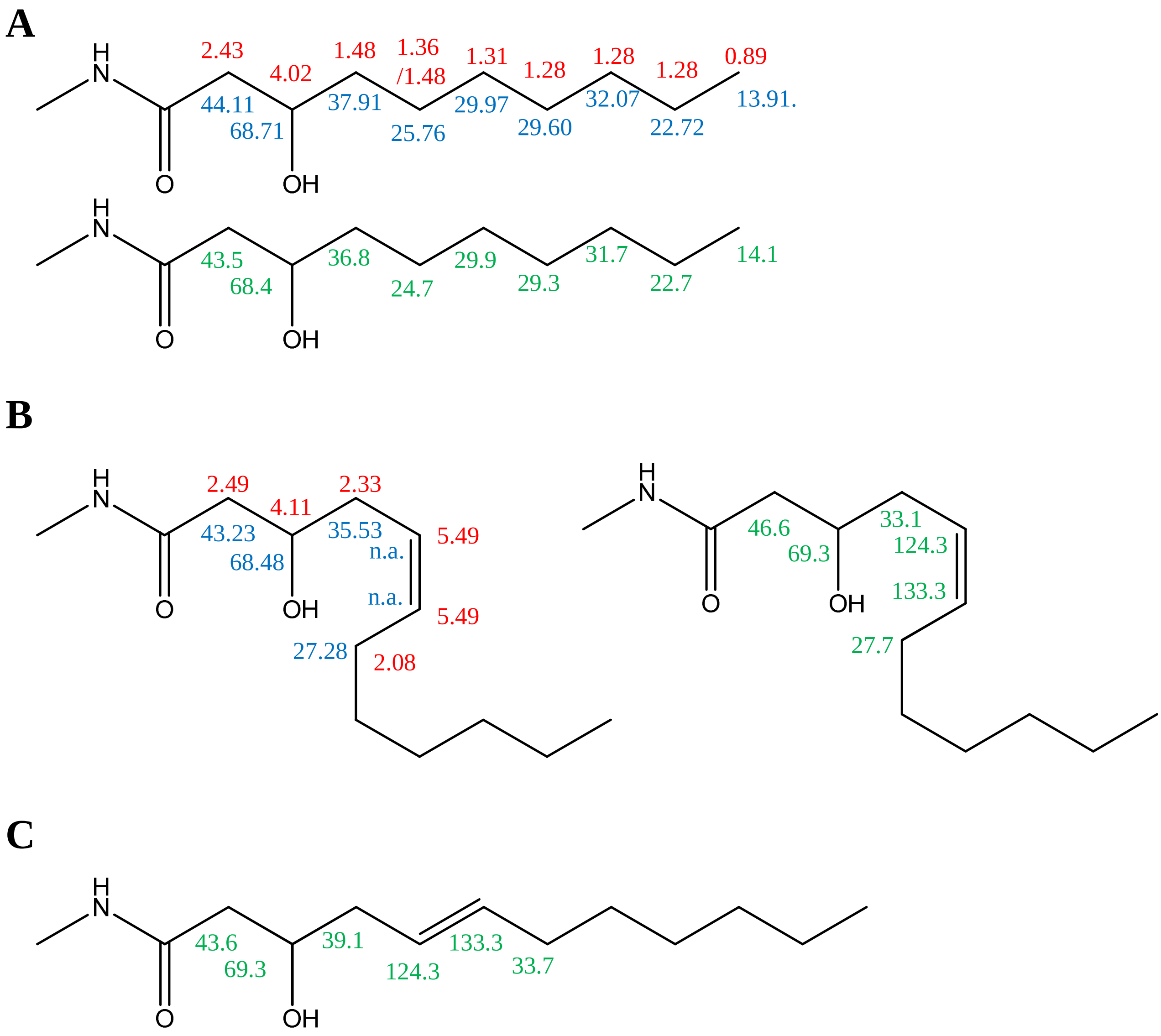

Figure S17. NMR analysis of fatty acid moiety in xantholysin C. ^{1}H and ^{13}C chemical shift comparison between the lipid tail of xantholysin A and variant 2. (A) Experimental ^{1}H (red) and ^{13}C (blue) chemical shifts of the xantholysin 3-hydroxydecanoic acid moiety, compared to predicted ^{13}C chemical shifts (green). (B) Experimental ^{1}H (red) and ^{13}C (blue) chemical shifts of the xantholysin variant 2 3-hydroxydodec-5-enoic acid moiety, compared to predicted ^{13}C chemical shifts (green) assuming a *cis*-configuration. (C) Predicted ^{13}C (green) chemical shifts of the xantholysin variant 2 3-hydroxydodec-5-enoic acid moiety assuming a *trans*-configuration. ^{13}C chemical shift predictions were calculated using ChemNMR Pro 12.0 as implemented in the ChemDraw Ultra 12.0 software (*PerkinElmer, Inc.*).

In the case of *cis*-3-hydroxydodec-5-enoic acid, the predicted ^{13}C chemical shifts indicate a decrease in both the γ and ζ ^{13}C chemical shifts compared to the HDA moiety (36.8 ppm→33.1 ppm and 29.3 ppm→27.7 ppm respectively). In the case of the *trans*-3-hydroxydodec-5-enoic acid, an increase in these same chemical shifts is predicted (36.8 ppm→39.1 ppm and 29.3 ppm→33.7 ppm respectively). In the *cis* configuration, the van der Waals radii of the hydrogens of the two carbons

flanking the double bond are overlapping due to their spatial proximity, as opposed to the trans configuration. This steric compression causes an additional shielding and thus upfield shift of the associated carbons, which is not present in the case of the trans configuration (for reference, see Breitmaier E., Voelter E. (1987) Carbon-13 NMR Spectroscopy: High-Resolution Methods and Applications in Organic Chemistry and Biochemistry, 3rd edition. Weinheim: VCH Verlagsgesellschaft mbH. 515 p.). The experimental values of the 3-hydroxydodec-5-enoic acid moiety compared to those of the HDA moiety (37.91 ppm→35.53 ppm and 29.60 ppm→27.28 ppm for the γ and ζ ^{13}C nuclei respectively) show that the ^{13}C chemical shifts correspond significantly better to the case of the *cis*-configuration, indeed showing a decrease. Therefore, this provides a significant indication for the lipid tail in xantholysin variant 2 to be in the *cis*-configuration.
