# Supplementary material for: The Antimicrobial Compound Xantholysin Defines a New Group of Pseudomonas Cyclic Lipopeptides
Source: PLoS One. 2013 May 17;8(5):e62946. doi: 10.1371/journal.pone.0062946 (PMC3656897; doi:10.1371/journal.pone.0062946)
Supplement: Table S1 — Characterization of antibacterial activity of P. putida BW11M1. Homology of genes disrupted in BW11M1 plasposon mutants lacking antibacterial activity using X. alfalfae subsp. alfalfae LMG 497 as indicator strain. (PDF) [file pone.0062946.s018.pdf]

Table S1. Characterization of antibacterial activity of *P. putida* BW11M1. Homology of genes disrupted in *P. putida* BW11M1 plasposon mutants lacking antibacterial activity using *X. alfalfae* subsp. *alfalfae* LMG 497 as indicator strain.

Mutant	Homology				
	Protein name	Accession nr	Function of gene product	Identity[a]	Reference[*]
CMPG2178, CMPG2179, CMPG2180, CMPG2181, CMPG2182, CMPG2183, CMPG2184	EtlA (PSEEN3332)	YP_608877	Entolysin synthetase A	75%	[1]
CMPG2185, CMPG2186, CMPG2187, CMPG2188, CMPG2189, CMPG2190, CMPG2191, CMPG2192, CMPG2193, CMPG2194	EtlB (PSEEN3045)	YP_608606	Entolysin synthetase B	69%	[1]
CMPG2195, CMPG2196, CMPG2197, CMPG2198, CMPG2199, CMPG2200	EtlC (PSEEN3044)	YP_608605	Entolysin synthetase C	72%	[1]
CMPG2201	EtlR (PSEEN3335)	YP_608878	Entolysin transcriptional regulator (LuxR family)	75%	[1]
CMPG2202	FahA (PSEEN4611)	YP_610068	Fumarylacetoacetase (homogentisate catabolism)	97%	[2]
CMPG2203	CysG (PSEEN2217)	YP_607840	Siroheme synthase	96%	[3]
CMPG2204	RecB (PSEEN0692)	YP_606434	Exonuclease V, beta subunit	90%	[4]
CMPG2205	ClpB (PSEEN4678)	YP_610130	ATP-dependent Clp protease, ATP-binding subunit	99%	[5]
CMPG2206	PSEEN0141	YP_605934	Surface adhesion protein LapA homologue	96%	[6]
CMPG2207	PSEEN3459	YP_608995	Alginate *O*-acetylation protein (AlgJ-like)	84%	[7]
CMPG2208	PA3156	NP_251846	LPS *N*-acetyltransferase WbpD homologue	84%	[8]
CMPG2209	PyrE (PSEEN5436)	YP_610831	Orotate phosphoribosyl-transferase	100%	[9]
CMPG2210	LeuS (PSEEN4813)	YP_610252	Leucyl-tRNA synthetase	98%	[10]
CMPG2211	PSEEN4860	YP_610295	Hypothetical protein	80%	[11]

[a] Amino acid sequence identity of the predicted full-length gene product deduced from the corresponding gene identified in the *P. putida* BW11M1 draft genomic sequence (unpublished data). For all mutants genes, highest homology was found for *Pseudomonas entomophila* L48 gene products [12], except for CMPG2208 with a disrupted gene encoding a homologue of *Pseudomonas aeruginosa* PAO1 WbpD.

* References

1. Vallet-Gely I, Novikov A, Augusto L, Liehl P, Bolbach G, et al. (2010) Association of hemolytic activity of *Pseudomonas entomophila*, a versatile soil bacterium, with cyclic lipopeptide production. Appl Environ Microbiol 76: 910–921.
2. Arias-Barrau E, Olivera ER, Luengo JM, Fernández C, Galán B, et al. (2004) The homogentisate pathway: a central catabolic pathway involved in the degradation of L-phenylalanine, L-tyrosine, and 3-hydroxyphenylacetate in *Pseudomonas putida*. J Bacteriol 186: 5062–5077.
3. Warren MJ, Roessner CA, Santander PJ, Scott AI (1990) The *Escherichia coli cysG* gene encodes S-adenosylmethionine-dependent uroporphyrinogen III methylase. Biochem J 265: 725–729.
4. Pavankumar TL, Sinha AK, Ray MK (2010) All three subunits of RecBCD enzyme are essential for DNA repair and low-temperature growth in the Antarctic *Pseudomonas syringae* Lz4W. PLoS One 5: e9412.
5. Weibezahn J, Tessarz P, Schlieker C, Zahn R, Maglica Z, et al. (2004) Thermotolerance requires refolding of aggregated proteins by substrate translocation through the central pore of ClpB. Cell 119: 653–665.
6. Hinsa SM, Espinosa-Urgel M, Ramos JL, O'Toole GA (2003) Transition from reversible to irreversible attachment during biofilm formation by *Pseudomonas fluorescens* WCS365 requires an ABC transporter and a large secreted protein. Mol Mcrobiol 49: 905–918.
7. Franklin MJ, Ohman DE (2002) Mutant analysis and cellular localization of the AlgI, AlgJ, and AlgF proteins required for O acetylation of alginate in *Pseudomonas aeruginosa*. J Bacteriol 184: 3000–3007.
8. Wenzel CQ, Daniels C, Keates RAB, Brewer D, Lam JS (2005) Evidence that WbpD is an N-acetyltransferase belonging to the hexapeptide acyltransferase superfamily and an important protein for O-antigen biosynthesis in *Pseudomonas aeruginosa* PAO1. Mol Microbiol 57: 1288–1303.
9. Henriksen A, Aghajari N, Jensen KF, Gajhede M (1996) A flexible loop at the dimer interface is a part of the active site of the adjacent monomer of *Escherichia coli* orotate phosphoribosyltransferase. Biochemistry 35: 3803–3809.
10. Liu Y, Liao J, Zhu B, Wang E-D, Ding J (2006) Crystal structures of the editing domain of *Escherichia coli* leucyl-tRNA synthetase and its complexes with Met and Ile reveal a lock-and-key mechanism for amino acid discrimination. Biochem J 394: 399–407.
11. Demirci H, Gregory ST, Dahlberg AE, Jogl G (2008) Multiple-site trimethylation of ribosomal protein L11 by the PrmA methyltransferase. Structure 16: 1059–1066.
12. Vodovar N, Vallenet D, Cruveiller S, Rouy Z, Barbe V, et al. (2006) Complete genome sequence of the entomopathogenic and metabolically versatile soil bacterium *Pseudomonas entomophila*. Nat Biotechnol 24: 673–679.
