# Supplementary material for: The Antimicrobial Compound Xantholysin Defines a New Group of Pseudomonas Cyclic Lipopeptides
Source: PLoS One. 2013 May 17;8(5):e62946. doi: 10.1371/journal.pone.0062946 (PMC3656897; doi:10.1371/journal.pone.0062946)
Supplement: Table S2 — In silico analysis of adenylation domains in xantholysin synthetases. Predicted substrate specificity of the A domains in the xantholysin NRPSs. (PDF) [file pone.0062946.s019.pdf]

Table S2. *In silico* analysis of adenylation domains in xantholysin synthetases. Predicted substrate specificity of the A domains in the xantholysin NRPSs.

		NRPSpredictor2			
**Protein**	**Domain**	**Signature**	**Motif**	**AA (score)**	**Phylotree[a]**
XtlA	A1	LWHAFDAMAWEPFLLVG GDINNYGPTEATVVASA	DAWFLGNVVK	Leu (100%)	Leu
	A2	LWGAFDGSVWEWKFFCG GGHNVYGPTEATVDSTA	DGWKFGVVDK	Asp (70%)	Glu
XtlB	A3	LWATFDASVWEWQFVCG GGHNVYGPTETTVDCTV	DAWQVGVVDK	Gln (70%)	Gln
	A4	LNNSFDASTLEAWLMVG GDLNGYGPTEATTFSTT	DALWMGGTFK	Val (80%)	Val
	A5	LWHAFDAMAWEPFLLVG GDVNNYGPTETTVVATS	DAWFLGNVVK	Leu (100%)	Leu/Ile
	A6	LWSTFDASVWEWQFVCG GGHNVYGPTETTVDCSV	DAWQVGVVDK	Gln (70%)	Gln
	A7	RWMTFDVSVWEWHFLCS GEHNLYGPTEAAVDVSA	DVWHLSLVDK	Ser (80%)	Ser
	A8	LNNSFDASTLEAWLMVG GDLNGYGPTEATTFSTT	DALWMGGTFK	Val (80%)	Val
	A9	LWHAFDAMAWEPFLLVG GDVNNYGPTETTVVATS	DAWFLGNVVK	Leu (100%)	Leu/Ile
	A10	LWSTFDASVWEWQFVCG GGHNVYGPTETTVDCSV	DAWQVGVVDK	Gln (70%)	Gln
XtlC	A11	LWHAFDAMAWEPFLLIG GDVNNYGPTETTVVASS	DAWFLGNVVK	Leu (100%)	Leu
	A12	LWHAFDAMAWEPFLLIG GDVNNYGPTEATVVATS	DAWFLGNVVK	Leu (100%)	Leu
	A13	LWSTFDASVWEWQFVCG GGHNVYGPTEATVDCSV	DAWQVGVVDK	Gln (70%)	Gln
	A14	LNNSFDAATLDGFVLCG GEVHCYGPTETTTFATA	DALFLGCTFK	Leu (70%)	Ile/Val/Leu

[a] Deduced from A domain clustering in Fig. 3
