# Supplementary material for: The Antimicrobial Compound Xantholysin Defines a New Group of Pseudomonas Cyclic Lipopeptides
Source: PLoS One. 2013 May 17;8(5):e62946. doi: 10.1371/journal.pone.0062946 (PMC3656897; doi:10.1371/journal.pone.0062946)
Supplement: Table S3 — Antagonistic activity spectrum of P. putida BW11M1 wild type (WT) and xantholysin-deficient mutants. The indicators shown are listed in Table 1 and Table S4. All other bacteria listed in Table S4 are not inhibited by strain BW11M1. Antagonism was semi-quantified by measuring the halo radii (in mm; average of three repeats; ND, no halo detected). Values in parentheses denote formation of a turbid halo. For some wild-type Xanthomonas strains a larger turbid halo was observed around a smaller clear halo. Additional italicized values in parentheses refer to the presence of a ‘secondary’ turbid growth inhibition halo. (PDF) [file pone.0062946.s020.pdf]

Table S3. Antagonistic activity spectrum of *P. putida* BW11M1 wild type (WT) and xantholysin-deficient mutants. The indicators shown are listed in Table 1 and Table S4. All other bacteria listed in Table S4 are not inhibited by strain BW11M1. Antagonism was semi-quantified by measuring the halo radii (in mm; average of three repeats; ND, no halo detected). Values in parentheses denote formation of a turbid halo. For some wild-type *Xanthomonas* strains a larger turbid halo was observed around a smaller clear halo. Additional italicized values in parentheses refer to the presence of a 'secondary' turbid growth inhibition halo.

Strain	WT	*xtlA*	*xtlB*	*xtlC*	*xtlR*
**α-Proteobacteria**					
Azospirillum brasilense Sp7	(5.0)	(3.0)	(3.0)	(3.0)	(3.0)
Sphingomonas wittichii RW1	9.5	4.0	4.0	4.0	4.0
**β-Proteobacteria**					
Burkholderia vietnamensis LMG 10927	3.5	ND	ND	ND	ND
Variovorax paradoxus LMG 1797	11.0	5.5	5.0	5.5	5.5
**γ-Proteobacteria**					
Aeromonas hydrophilia ATCC 7966	4.5	ND	ND	ND	ND
Erwinia amylovora CFBP 1430	(4.5)	ND	ND	ND	ND
Xanthomonas alfalfae subsp. *alfalfae* LMG 497	2.5	ND	ND	ND	ND
X. axonopodis pv. *manihotis* LMG 784	3.0	ND	ND	ND	ND
X. campestris pv. *campestris* LMG 582	1.0	ND	ND	ND	ND
X. citri pv. *malvacearum* LMG 761	1.5	ND	ND	ND	ND
X. hortorum pv. *hederae* LMG 7411	11.5	8.5	8.0	8.5	8.5
X. sacchari LMG 471	3.5	ND	ND)	ND	ND
	(12.5)	*(9.0)*	*(9.5)*	*(9.0)*	*(9.5)*
X. translucens LMG 12921	1.5	ND	ND	ND	ND
X. translucens pv. *cerealis* LMG 679	3.0	ND	ND	ND	ND
	(15.0)	*(10.0)*	*(9.5)*	*(10.0)*	*(10.0)*
X. translucens pv. *graminis* LMG 726	(13.5)	(10.0)	(9.5)	(10.0)	(9.5)
X. translucens pv. *hordei* LMG 737	10.5	7.5	8.0	7.5	7.5
X. vasicola pv. *holcicola* LMG 736	8.5	5.0	5.5	5.0	5.5
X. vasicola pv. *musacearum* LMG 785	3.0	ND	ND	ND	ND
	(28.5)	*(18.5)*	*(18.5)*	*(18.5)*	*(18.5)*
X. vasicola pv. *musacearum* LMG 7431	2.5	ND	ND	ND	ND
	(24.0)	*(17.0)*	*(17.5)*	*(17.0)*	*(17.0)*
Xanthomonas sp. pv. *zinnia* LMG 8692	3.0	ND	ND	ND	ND
**Actinobacteria**					
Mycobacterium smegmatis DSM 43756	6.0	3.5	3.5	3.5	4.0
Rhodococcus erythropolis N11	4.0	2.0	1.5	1.5	1.5
Streptomyces coelicolor A3(2)	6.0	2.5	2.0	2.0	2.5
Streptomyces lividans TK24	6.5	3.0	3.0	3.0	3.0
**Firmicutes**					
Bacillus megaterium ATCC 13632	19.0	9.0	8.5	8.0	8.5
Bacillus subtilis LMG 7135	7.5	5.0	5.0	4.5	4.5
Staphylococcus aureus ATCC 6358	9.5	5.0	5.0	5.0	5.0
