# Supplementary material for: The Antimicrobial Compound Xantholysin Defines a New Group of Pseudomonas Cyclic Lipopeptides
Source: PLoS One. 2013 May 17;8(5):e62946. doi: 10.1371/journal.pone.0062946 (PMC3656897; doi:10.1371/journal.pone.0062946)
Supplement: Table S4 — Analysis of bacterial susceptibility to P. putida BW11M1. List of non-Xanthomonas bacteria used as indicators. (PDF) [file pone.0062946.s021.pdf]

Table S4. Analysis of bacterial susceptibility to *P. putida* BW11M1. List of non-*Xanthomonas* bacteria used as indicators.

Bacterial strain	Source or reference*
**Gram-negative bacteria**	
**α-Proteobacteria**	
Agrobacterium tumefaciens A208	[1]
Azospirillum brasilense Sp7	[2]
Sphingomonas wittichii RW1	[3]
**β-Proteobacteria**	
Bordetella avium 197N	[4]
Burkholderia cepacia LMG 1222	BCCM[a]
Burkholderia vietnamiensis LMG 10927	BCCM
Variovorax paradoxus LMG 1797	BCCM
**γ-Proteobacteria**	
Aeromonas hydrophila ATCC 7966	ATCC[b]
Citrobacter freundii ATCC 8090	ATCC
Enterobacter aerogenes ATCC 13048	ATCC
Erwinia amylovora CFBP 1430	CFBP[c]
Klebsiella pneumoniae ATCC 13883	ATCC
Proteus vulgaris LMM 2011	LMM[d]
Salmonella enteritidis ATCC 13076	ATCC
Serratia entomophila DSM 12358	DSMZ[e]
Shigella flexneri LMG 10472	BCCM
Yersinia enterocolitica LMG 7899	BCCM
**Gram-positive bacteria**	
**Firmicutes**	
Lactobacillus plantarum LMG-P21295	[5]
Lactobacillus rhamnosus GG (LMG 6400)	BCCM
Bacillus megaterium ATCC 13632	ATCC
Bacillus subtilis LMG 7135	BCCM
Staphylococcus aureus ATCC 6358	ATCC
**Actinobacteria**	
Rhodococcus erythropolis N11	[6]
Mycobacterium smegmatis DSM 43756	DSMZ
Streptomyces coelicolor A3(2)	DSMZ
Streptomyces lividans TK24	[7]

[a] BCCM, Belgian Coordinated Collections of Microorganisms

[b] ATCC, American Type Culture Collection

[c] CFBP: French Collection of Plant-associated Bacteria

[d] LMM: Laboratory of Food Microbiology, KU Leuven

[e] DSMZ: Leibniz Institute DSMZ-German Collection of Microorganisms and Cell Cultures

* References

1. Wirawan IG, Kang HW, Kojima M (1993) Isolation and characterization of a new chromosomal virulence gene of *Agrobacterium tumefaciens*. J Bacteriol 175: 3208–3212.

2. De Mot R, Vanderleyden J (1989) Application of two-dimensional protein analysis for strain fingerprinting and mutant analysis of *Azospirillum* species. Can J Microbiol 35: 960–967.

3. Miller TR, Delcher AL, Salzberg SL, Saunders E, Detter JC, et al. (2010) Genome sequence of the dioxin-mineralizing bacterium *Sphingomonas wittichii* RW1. J Bacteriol 192: 6101–6102.

4. Sebaihia M, Preston A, Maskell DJ, Kuzmiak H, Connell TD, et al. (2006) Comparison of the genome sequence of the poultry pathogen *Bordetella avium* with those of *B. bronchiseptica*, *B. pertussis*, and *B. parapertussis* reveals extensive diversity in surface structures associated with host interaction. J Bacteriol 188: 6002–6015.

5. Ström K, Sjögren J, Broberg A, Schnürer J (2002) *Lactobacillus plantarum* MiLAB 393 produces the antifungal cyclic dipeptides cyclo(L-Phe-L-Pro) and cyclo(L-Phe-trans-4-OH-L-Pro) and 3-phenyllactic acid. Appl Environ Microbiol 68: 4322–4327.

6. Nagy I, Compernolle F, Ghys K, Vanderleyden J, De Mot R (1995) A single cytochrome P-450 system is involved in degradation of the herbicides EPTC (*S*-ethyl dipropylthiocarbamate) and atrazine by *Rhodococcus* sp. strain NI86/21. Appl Environ Microbiol 61: 2056–2060.

7. Simonet JM, Boccard F, Pernodet JL, Gagnat J, Guérineau M (1987) Excision and integration of a self-transmissible replicon of *Streptomyces ambofaciens*. Gene 59: 137–144.
