# Supplementary material for: The Antimicrobial Compound Xantholysin Defines a New Group of Pseudomonas Cyclic Lipopeptides
Source: PLoS One. 2013 May 17;8(5):e62946. doi: 10.1371/journal.pone.0062946 (PMC3656897; doi:10.1371/journal.pone.0062946)
Supplement: Table S5 — Antifungal activity of P. putida BW11M1 and xantholysin-deficient mutants. The effect on mycelial growth near a bacterial colony is indicated: (+) inhibition by wild type; (↓) or (−), reduced or no inhibition by xtl mutants compared to WT (as observed on PTA medium). For a particular indicator, the same phenotype was observed for the xtlA, xtlB, xtlC and xtlR mutants (CMPG2183, CMPG2187, CMPG2198, and CMPG2201, respectively), collectively designated xtl mutants. The inhibitory patterns for selected fungal strains are shown in Fig. S5. (PDF) [file pone.0062946.s022.pdf]

Table S5. Antifungal activity of *P. putida* BW11M1 and xantholysin-deficient mutants. The effect on mycelial growth near a bacterial colony is indicated: (+) inhibition by wild type; (↓) or (-), reduced or no inhibition by *xtl* mutants compared to WT (as observed on PTA medium). For a particular indicator, the same phenotype was observed for the *xtlA*, *xtlB*, *xtlC* and *xtlR* mutants (CMPG2183, CMPG2187, CMPG2198, and CMPG2201, respectively), collectively designated *xtl* mutants. The inhibitory patterns for selected fungal strains are shown in Fig. S5.

**Fungal strain**	**Growth inhibition**	
	Wild type	*xtl* mutants
Alternaria longipes CBS 620.83	+	-
Alternaria porri (CMPG collection)	+	NT[a]
Ascochyta pisi MUCL30164	+	↓
Aspergillus flavus CBS111.45	+	-
Botrytis cinerea JHCC8973	+	-
Botrytis cinerea MUCL30158	+	-
Colletotrichum gloeosporoides SR24	+	↓
Fusarium culmorum MUCL30162	+	-
Fusarium graminearum MUCL30161	+	-
Fusarium oxysporum MUCL909	+	-
Fusarium oxysporum MUCL30160	+	-
Fusarium oxysporum f. sp. *radicis-lycopersi* CBS 873.95	+	-
Fusarium oxysporum f. sp. *vasinfectum* CBS 410.90	+	-
Gloeosporium musarum MUCL500	+	-
Gloeosporium solani CBS 194.32	+	-
Nectria haematococca MUCL20259	+	-
Neurospora crassa (CMPG collection)	+	-
Penicillium expansum (CMPG collection)	+	-
Pyricularia oryzae (CMPG collection)	+	↓
Pyrenophora tritici-repentis MUCL30217	+	↓
Rhizoctonia solani CBS 211.84	+	-
Rhizoctonia solani NL84	+	NT
Septoria nodorum MUCL30111	+	-
Trichoderma viride MUCL29726	+	-
Verticillium albo-atrum MUCL19212	+	-
Verticillium dahliae MUCL 19210	+	-

[a] Not tested
