# Supplementary material for: The Antimicrobial Compound Xantholysin Defines a New Group of Pseudomonas Cyclic Lipopeptides
Source: PLoS One. 2013 May 17;8(5):e62946. doi: 10.1371/journal.pone.0062946 (PMC3656897; doi:10.1371/journal.pone.0062946)
Supplement: Table S6 — NMR analysis of xantholysin A. 1H and 13C NMR assignment of xantholysin A in DMF-d7 solution, 55°C. (PDF) [file pone.0062946.s023.pdf]

Table S6. NMR analysis of xantholysin A. ^{1}H and ^{13}C NMR assignment of xantholysin A in DMF-d7 solution, 55°C.

			^{1}H δ [ppm]	^{13}C δ [ppm]				^{1}H δ [ppm]	^{13}C δ [ppm]
**HDA**		CO		173.28	**Gln3**				
		CH_2 α	2.43	44.11	$^{3}J_{HNH\alpha}$ [Hz]	6.14	NH	8.34	
		CH β	4.02	68.71			CH α	4.26	55.64
		CH_2 γ	1.48	37.91			CO		n.a.
		CH_2 δ1	1.36	25.76			CH_2 β	2.13	27.17
		CH_2 δ2	1.48	25.76			CH_2 γ	2.38	32.38
		CH_2 ε	1.31	29.97			CO δ		n.a.
		CH_2 ζ	1.28	29.60			NH_2	6.71/7.40	
		CH_2 η	1.28	32.07	**Val4**				
		CH_2 θ	1.28	22.72	$^{3}J_{HNH\alpha}$ [Hz]	6.93	NH	7.70	
		CH_3 ι	0.88	13.91			CH α	4.02	61.85
		OH	n.a.				CO		n.a.
**Leu1**							CH β	2.26	29.93
$^{3}J_{HNH\alpha}$ [Hz]	6.50	NH	8.30				CH_3 γ	0.96	19.22
		CH α	4.30	52.87			CH_3 γ	1.01	19.46
		CO		n.a.	**Leu5**				
		CH_2 β1	1.67	40.96	$^{3}J_{HNH\alpha}$ [Hz]	n.d.	NH	7.95	
		CH_2 β2	1.74	40.96			CH α	4.18	54.65
		CH γ	1.74	24.85			CO		n.a.
		CH_3 δ	0.91	21.80			CH_2 β	1.71	40.39
		CH_3 δ	0.95	23.13			CH γ	1.74	24.97
**Glu2**							CH_3 δ	0.89	21.26
$^{3}J_{HNH\alpha}$ [Hz]	ca 4.5	NH	8.72				CH_3 δ	0.94	22.63
		CH α	4.27	55.78	**Gln6**				
		CO		174.31	$^{3}J_{HNH\alpha}$ [Hz]	6.58	NH	8.24	
		CH_2 β1	2.04	27.02			CH α	4.09	53.91
		CH_2 β2	2.15	27.02			CO		172.54
		CH_2 γ	2.45	31.38			CH_2 β	2.05	27.08
		CO δ		n.a.			CH_2 γ	2.28	32.18
		OH	n.a.				CO δ		n.a.
							NH_2	6.55/7.19	

n.d. = not determined
n.a. = not assigned

Table S6. NMR analysis of xantholysin A. ^{1}H and ^{13}C NMR assignment of xantholysin A in DMF-d7 solution, 55°C (continued).

			^{1}H δ [ppm]	^{13}C δ [ppm]				^{1}H δ [ppm]	^{13}C δ [ppm]
**Ser7**					**Leu11**				
$^{3}J_{HNH\alpha}$ [Hz]	5.32	NH	7.89		$^{3}J_{HNH\alpha}$ [Hz]	n.d.	NH	7.95	
		CH α	4.58	54.68			CH α	4.32	53.62
		CO		172.14			CO		173.51
		CH_2 β1	4.38	64.01			CH_2 β1	1.60	41.18
		CH_2 β2	4.50	64.01			CH_2 β2	1.81	41.18
**Val8**							CH γ	1.83	24.94
$^{3}J_{HNH\alpha}$ [Hz]	6.79	NH	8.20				CH_3 δ	0.90	21.92
		CH α	4.09	61.90			CH_3 δ	0.94	22.91
		CO		173.90	**Leu12**				
		CH β	2.40	30.25	$^{3}J_{HNH\alpha}$ [Hz]	7.12	NH	7.79	
		CH_3 γ	0.98	19.50			CH α	4.34	53.23
		CH_3 γ	1.08	19.12			CO		173.76
**Leu9**							CH_2 β	1.71	40.64
$^{3}J_{HNH\alpha}$ [Hz]	n.d.	NH	8.03				CH γ	1.76	25.02
		CH α	4.20	54.48			CH_3 δ	0.90	21.61
		CO		174.04			CH_3 δ	0.93	23.33
		CH_2 β1	1.59	40.28	**Gln13**				
		CH_2 β2	1.84	40.28	$^{3}J_{HNH\alpha}$ [Hz]	6.70	NH	7.25	
		CH γ	1.83	25.02			CH α	4.25	54.27
		CH_3 δ	0.87	21.10			CO		172.30
		CH_3 δ	0.92	23.05			CH_2 β1	1.95	27.32
**Gln10**							CH_2 β2	2.32	27.31
$^{3}J_{HNH\alpha}$ [Hz]	6.84	NH	7.74				CH_2 γ	2.22	32.22
		CH α	4.33	54.43			CO δ		n.a.
		CO		172.98			NH_2	6.63/7.19	
		CH_2 β1	2.04	27.25	**Ile14**				
		CH_2 β2	2.18	27.25	$^{3}J_{HNH\alpha}$ [Hz]	8.51	NH	7.48	
		CH_2 γ	2.38	32.48			CH α	4.37	57.47
		CO δ		n.a.			CO		n.a.
		NH_2	6.55/7.19				CH β	1.89	37.06
							CH_3 γ	0.88	15.82
							CH_2 γ1	1.19	25.30
							CH_2 γ2	1.41	25.30
							CH_3 δ	0.85	11.00

n.d. = not determined
n.a. = not assigned
